# Hydrogels Based on Proteins Cross-Linked with Carbonyl Derivatives of Polysaccharides, with Biomedical Applications

**DOI:** 10.3390/ijms25147839

**Published:** 2024-07-17

**Authors:** Chahrazed Mahmoudi, Naïma Tahraoui Douma, Hacene Mahmoudi, Camelia Elena Iurciuc (Tincu), Marcel Popa

**Affiliations:** 1 Laboratory of Water and Environment, Faculty of Technology, University Hassiba Benbouali of Chlef, Chlef 02000, Algeria; 2 Department of Natural and Synthetic Polymers, Faculty of Chemical Engineering and Protection of the Environment, “Gheorghe Asachi” Technical University, 700050 Iasi, Romania; 3 National Higher School of Nanosciences and Nanotechnologies, Algiers 16000, Algeria; hac.mahmoudi@gmail.com; 4 Department of Pharmaceutical Technology, Faculty of Pharmacy, “Grigore T. Popa” University of Medicine and Pharmacy, University Street, No. 16, 700115 Iasi, Romania; 5 Academy of Romanian Scientists, 3 Ilfov, 050044 Bucharest, Romania

**Keywords:** polysaccharides, carbonyl groups, hydrogels, Schiff base, biomedical applications

## Abstract

Adding carbonyl groups into the hydrogel matrix improves the stability and biocompatibility of the hydrogels, making them suitable for different biomedical applications. In this review article, we will discuss the use of hydrogels based on polysaccharides modified by oxidation, with particular attention paid to the introduction of carbonyl groups. These hydrogels have been developed for several applications in tissue engineering, drug delivery, and wound healing. The review article discusses the mechanism by which oxidized polysaccharides can introduce carbonyl groups, leading to the development of hydrogels through cross-linking with proteins. These hydrogels have tunable mechanical properties and improved biocompatibility. Hydrogels have dynamic properties that make them promising biomaterials for various biomedical applications. This paper comprehensively analyzes hydrogels based on cross-linked proteins with carbonyl groups derived from oxidized polysaccharides, including microparticles, nanoparticles, and films. The applications of these hydrogels in tissue engineering, drug delivery, and wound healing are also discussed.

## 1. Introduction

The term “hydrogel” refers to a three-dimensional, hydrophilic network capable of absorbing and retaining large amounts of water. Despite their high water content, hydrogels generally exhibit solid-like behavior [1]. These swollen polymer networks create environments that closely mimic certain aspects of living systems, making them highly popular in various fields, including medical, pharmaceutical, food, agriculture, and environmental applications [1,2]. Hydrogels are valued for their polymeric nature and for meeting specific criteria, such as the capacity to retain water, inertness, biocompatibility, photostability, and sufficient permeability to biological fluids. These properties enable hydrogels to enhance existing products and develop new formulations that address human health issues and improve the overall quality of life [3,4].

Polysaccharides are complex carbohydrates composed of long chains of monosaccharide units connected by glycosidic bonds with high molecular weights [5]. They are found in nature, originating from microorganisms, algae, plants, and animals, and play critical roles in biological functions, such as cell wall formation and energy storage [6,7].

Their bioactive properties hold great potential in pharmaceuticals for therapeutic interventions [8]. In addition, their unique physicochemical properties make them valuable in the food [9,10], cosmetics [11], and materials science industries [12]. Ongoing research advances the understanding of their structure–function relationships, leading to innovative developments. In addition, researchers are modifying polysaccharides to improve their versatility and usability by changing the branching patterns of polymer chain lengths or adding functional groups and tailoring them to suit specific applications [13].

An essential method of modifying polysaccharides is introducing carbonyl groups into the biopolymer chain by oxidation [13,14]. This modification aims to improve the functionality and performance of polysaccharides for specific applications. By adding carbonyl groups, researchers intend to add new chemical and physical properties to the polysaccharide structure [15]. This modification can expand the potential utility of polysaccharides in various fields by influencing solubility, stability, and reactivity. Introducing a carbonyl functional group into the polysaccharide backbone is typically achieved using chemical oxidation methods [15,16,17]. This process controls molecular structure modification, allowing researchers to fine-tune the desired properties. As a result, the modified polysaccharides exhibit increased chemical reactivity, which makes them suitable for targeted functionalization and further customization. In biomedical applications, these customized polysaccharides have shown great potential for drug delivery systems, where controlled release and enhanced bioavailability are critical factors. In materials science, these tailored polysaccharides are also helpful in creating advanced biomaterials with improved mechanical strength and responsiveness to environmental stimuli. The incorporation of carbonyl groups in polysaccharides has been crucial in the development of hydrogel fabrication.

Adding carbonyl groups to polysaccharides enhances their ability to form hydrogels and provides distinct functionalities to the resulting materials. The carbonyl groups added to the polysaccharide structure increase its reactivity and cross-linking potential. This improved reactivity allows precise control over the hydrogel’s mechanical properties, swelling behavior, and responsiveness to external stimuli. In addition, introducing carbonyl groups makes it easier to include other functional molecules, such as amino groups from protein chains. Cross-linking of modified polysaccharides, particularly those with added carbonyl groups, with proteins is a significant method in biomaterials and biomedical research [18,19].

Proteins are natural polymers composed of amino acids linked by peptide bonds and cross-linked between chains by sulfhydryl bonds, hydrogen bonds, and van der Waals forces to form complex, functional structures [20]. Proteins, such as collagen, gelatin, albumin, and soy protein, are commonly used to create hydrogels [21]. The various functional groups present in proteins (e.g., amino, carboxyl, hydroxyl, and sulfhydryl groups) facilitate multiple types of interactions and cross-linking methods, making proteins an ideal choice for hydrogel formation [9].

Proteins, the fundamental building blocks of life, offer a rich diversity of chemical functionalities and structural motifs that are well-suited for creating advanced materials, like hydrogels. Harnessing proteins’ innate properties paves the way for developing hydrogels that promote cell growth and tissue regeneration and possess tailored mechanical properties and degradation rates [9]. Integrating protein-derived materials with hydrogel technology represents a frontier in biomaterials research, promising innovative solutions to complex challenges in healthcare and environmental sustainability.

Carbonyl-modified polysaccharides cross-linked with protein-based hydrogels represent a promising class of materials in biomaterials and biomedical applications. These hydrogels combine the advantageous properties of polysaccharides and proteins, offering unique characteristics, such as biocompatibility, biodegradability, and tunable mechanical properties. One of the primary cross-linking mechanisms in these hydrogels is Schiff base formation.

The Schiff base method is a commonly used technique for cross-linking. It involves introducing carbonyl groups into a modified polysaccharide backbone, which then reacts with amino groups in proteins to form covalent bonds called Schiff bases [8,22]. This reaction occurs through condensation between the polysaccharides’ carbonyl groups and the proteins’ amino groups, resulting in a stable cross-linked network. The Schiff base formation is often pH-dependent, and the reaction kinetics can be controlled to achieve the desired cross-linking degree [9]. The modified polysaccharides and proteins create a cross-linked structure, which imparts unique properties to the hydrogel. This hybrid hydrogel system combines the structural and mechanical features of polysaccharides with the bioactivity and functionality of proteins. The Schiff base reaction’s tunable nature allows precise control over the hydrogel’s physical and chemical characteristics. Schiff base hydrogels, characterized by their diverse compositions resulting from cross-linking modified polysaccharides with proteins, exhibit different shapes tailored to specific biomedical applications. Injectable liquid or semi-liquid hydrogels offer a minimally invasive approach to drug delivery and regenerative medicine [10,11]. Hydrogel films are thin, flexible sheets used for wound dressing and drug delivery, as they can conform to the body’s contours [11,13,14]. Spherical hydrogel micro/nanospheres enable targeted drug release, while macroscopic hydrogel structures support tissue engineering and organoid development [17,18]. Hydrogel nanoparticles are highly effective in delivering drugs and imaging agents to cells due to their nanoscale size [15]. Schiff base hydrogels have a wide range of biomedical applications. These hydrogels provide precise control over release kinetics in drug delivery, ensuring the best therapeutic outcomes. Tissue engineering benefits from the supportive environment offered by hydrogels, which facilitates cell growth and the regeneration of artificial tissues and organs. Hydrogel dressings are also used for wound healing, as they retain moisture, promote tissue regeneration, and prevent infections. Schiff base hydrogels come in diverse shapes and compositions, making them versatile in addressing various biomedical challenges and advancing innovative solutions in medicine and regenerative therapies [16,23,24].

This review focuses on the development and applications of hydrogels made from polysaccharides modified through oxidation by introducing carbonyl groups. The review article emphasizes the potential of oxidized polysaccharides containing carbonyl groups to cross-link proteins, resulting in a diverse class of hydrogels with adjustable mechanical properties and high biocompatibility. The following chapters explain the meticulous process of forming these hydrogels through protein cross-linking with carbonyl derivatives of polysaccharides, showing their dynamic properties and potential as biomaterials for various biomedical applications. The review concludes with a detailed analysis of the biomedical applications of these hydrogels, including their use in tissue engineering, drug delivery, and wound healing in various forms, such as microparticles, nanoparticles, and films.

## 2. Polysaccharides Modified by Oxidation for the Introduction of Carbonyl Groups

Polysaccharides are natural polymers that possess various physical properties and can serve as an excellent source of materials for many fields of application in the future. By developing their chemical modification, we can introduce hydrophilic, acidic, basic, or other functional groups into the structure of polysaccharides, thereby altering their properties [19]. Polysaccharides can be modified through chemical oxidation, an important method to produce structures that can be used for various applications. This method enhances product value by cleaving glycols oxidatively, chemically oxidizing primary alcohols to carboxylic acids, and enzymatically oxidizing primary alcohols to aldehydes [19]. These oxidation methods can modify the hydroxyl groups in the polysaccharide backbones to carboxyl or carbonyl groups. This modification can enhance the chain extensions and reactivity of the polysaccharides, leading to increased value for possible new applications [25]. Recently, some researchers reported that through selective oxidation of the hydroxyl group, carbonyl (aldehydes, CHO, and ketones, C=O) and carboxyl groups (COOH) are formed on the backbone of oxidized polysaccharides [19,26]. Hydroxyl groups on the primary C-6 position can be oxidized to form an aldehyde or a carboxylic acid [19]. The nitroxyl radical 2,2,6,6-tetramethyl piperidine-1-oxyl (TEMPO) is one of the most promising methods to produce polyuronic acids from polysaccharides by selective oxidation of primary alcohol (C-6) groups [27,28,29]. Jausovec et al. oxidized the cellulose nanofibers by a chemo-enzymatic modification using laccase as biocatalysts and TEMPO or 4-Amino-TEMPO as mediators under mild aqueous conditions (pH 5, 30 °C) to introduce surface-active aldehydes and carboxylic groups [30]. When using oxidizing agents, such as sodium hypochlorite and hydrogen peroxide, the proportion of dicarboxylic and dialdehyde functional groups can differ due to oxidation of the secondary hydroxyl groups (C-2 and C-3) [27,31,32].

### 2.1. Introduction of Carbonyl Groups in Polysaccharide Chains

The introduction of the carbonyl group in the backbone chain of polysaccharides is essential due to its critical impact on the structural and reactive properties. The main ways to create carbonyl groups on the polysaccharide chains are chemical and enzymatic oxidation [25]. These oxidation methods have been used to oxidize the hydroxyl group in the C-6 position, forming aldehyde and causing cleavage of vicinal diols at C-2 and C-3 to introduce dicarbonyl functional groups [19].

#### 2.1.1. Enzymatic Oxidation

**Figure 1:**
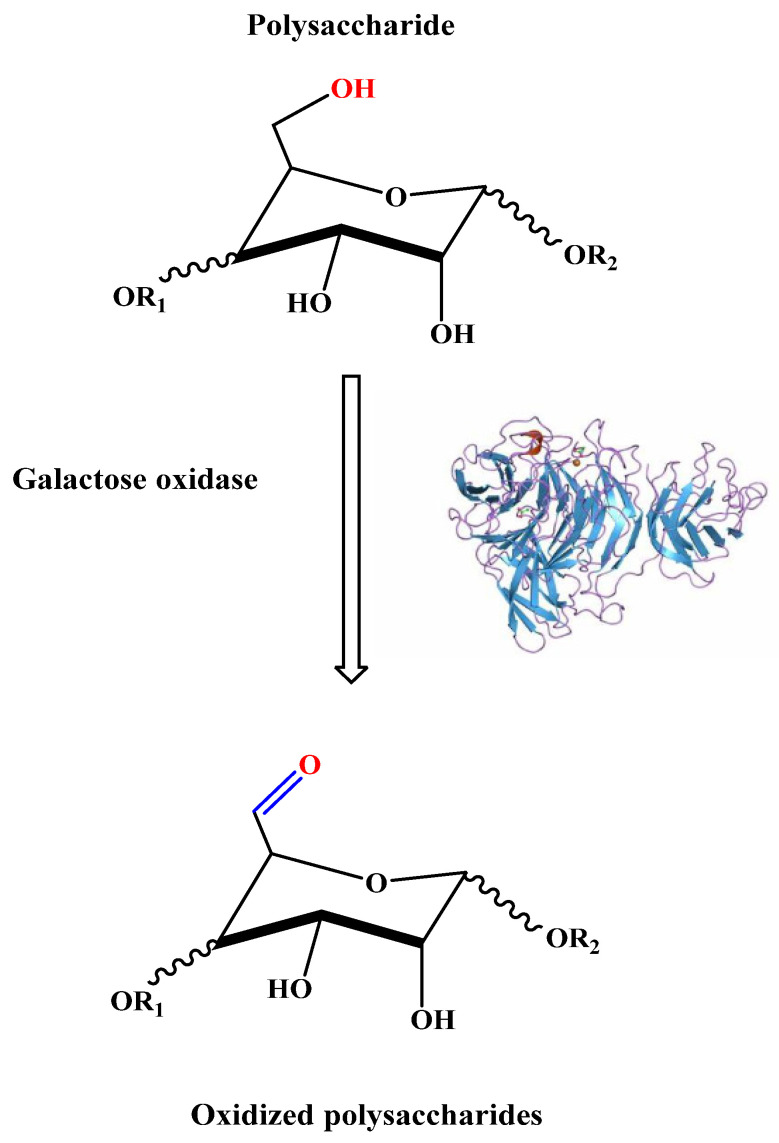
Schematic representation of enzymatic oxidation using galactose oxidase.

An enzymatic oxidation method is commonly used to modify and improve the properties of polysaccharides. Galactose oxidase (D-galactose: oxygen 6-oxidoreductase, E.C.1.1.3.9) is an enzyme that can be used instead of chemical catalysts. This enzyme selectively catalyzes the oxidation of the C-6 primary hydroxyl group of D-galactose moieties to produce corresponding aldehydes, as illustrated in Figure 1 [29]. The galactoaldehyde products are characterized by higher reactivity, which has been used for many applications, such as forming hydrogel cross-linking [33] and creating new aerogels [34]. The galactose oxidase catalyst’s enzymatic oxidation reaction occurs through oxidative and reductive half-reactions using oxygen as an oxidant, producing hydrogen peroxide as the reduced by-product [35,36].

The oxidation reaction conditions for oxidase-catalyzed galactose were optimized by Parikka et al. using the monosaccharide system methyl alpha-D-galactopyranoside. The optimal results were found with the combination of three enzymes (galactose oxidase, catalase, and horseradish peroxidase) in water, which gives an approximately 90% yield of the corresponding aldehyde. Uronic acid (methyl alpha-D-galactopyranosid uronic acid) and an alpha,beta-unsaturated aldehyde were produced as secondary products, which may result from the impurities from the preparation of galactose oxidase [37].

Parikka et al. used the selective oxidation of several galactose-containing polysaccharides, including spruce galactoglucomannan (O-acetyl galactoglucomannan), guar galactomannan (guar gum), larch arabinogalactan (corn arabinoxylan (corn fiber gum), and tamarind seed xyloglucan using the same three-enzyme system (galactose oxidase, catalase, and horseradish peroxidase). This study examined how temperature, substrate concentration, enzyme–substrate ratio, and polysaccharide structure affect the quantity and quality of the resulting product during oxidation. The results show that a higher oxidation degree was obtained in the case of oxidized O-acetyl galactoglucomannan, guar gum, and xyloglucan rather than in the oxidized larch arabinogalactan and corn fiber gum [29]. The oxidation of polysaccharides using galactose-6-oxidase was also reported in some earlier studies. Mollerup et al. use chemo-enzymatic methods with galactose-6-oxidase to oxidize galactoglucomann and xyloglucan units from polysaccharides. Oxidized polysaccharides can then be adsorbed onto cellulose surfaces to improve cellulose-based materials’ reactivity and barrier properties. This research aimed to develop this system for renewable energy and materials use [38].

Several studies have investigated the chemo-enzymatic modification of cellulosic pulps using the laccase-mediator system (LMS), which consists of laccase (EC 1.10.3.2) and the 2,2,6,6-tetramethylpiperidine-1-oxyl radical (TEMPO). This system operates under mild aqueous conditions (sodium citrate buffer, pH 6, 30 °C) and primarily introduces aldehyde groups into cellulose, with carboxyl groups forming a smaller proportion of the carbonyl groups. The reaction proceeds uniformly, even in the high-molecular-weight region, distinguishing it from other cellulose chemical oxidation methods. The effectiveness of the LMS in modifying cellulosic fibers has been demonstrated, particularly in softwood-derived cellulosic fibers (bleached softwood kraft pulp) [39]. By combining laccase and TEMPO, carboxyl and aldehyde groups can be introduced into the fibers, with process conditions optimized to achieve maximum increases in functional group contents. The efficacy of this system is attributed to catalytic oxidation pathways, offering insights into enzymatic modification for paper-making applications.

Furthermore, studies have shown that increasing pulp consistency enhances the biorefining potential of the laccase–TEMPO system. Higher pulp consistency leads to increased contents of aldehyde and carboxyl groups, along with more significant reductions in pulp viscosity during the enzyme treatment, affecting fiber strength and tear strength but also resulting in improved water retention values and mechanical properties, such as dry tensile index and burst index. The treatment enhances inter-fiber hydrogen bonding, leading to savings in refining energy and improved wet tensile strength. The increase in wet tensile strength is attributed to inter-fiber covalent bonding facilitated by aldehyde groups [40,41].

#### 2.1.2. Periodate Oxidation

Periodate oxidation in aqueous solutions is one of the most helpful modification methods to introduce carbonyl groups on the polysaccharides chain since it may entirely modify the physical and chemical properties of the oxidized biopolymers [42,43]. Usually, the periodate oxidation takes place in the presence of sodium metaperiodate as an oxidizing agent to attain the ring-opening of the 1,2-diols at C-2 and C-3 of polysaccharides to dialdehyde groups by high selectivity and efficiency [19]. Malaprade discovered the cleavage reaction by observing the rapid oxidation of polyols by the periodate ion [44,45]. Afterward, Criegee detected that lead tetraacetate cleaves 1,2 diols [46], but Fleury and Lange confirmed that the cleavage reaction of Malaprade succeeded based om the presence of vicinal hydroxyl groups in the compound [47]. The periodate oxidation was reviewed before [43,48] and will only be briefly commented on here.

**Figure 2:**
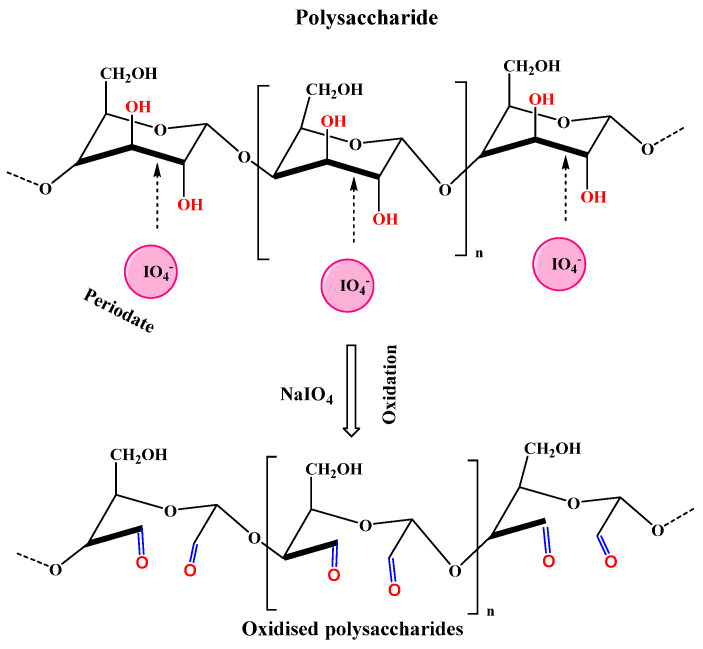
Schematic representation of oxidation using sodium periodate.

Figure 2 shows the mechanism for the periodate oxidation reaction. The process begins with the hydroxyls attaching directly to the iodine, occurring through two successive attacks of the ion pairs on each hydroxyl group. The result is the creation of a cyclic intermediate known as a periodate ester. Next, the cyclic iodate ester breaks down to form dialdehyde groups. The primary step in the mechanism is the generation of the ester periodate. The substrate’s structure, the reaction’s pH, and the temperature can influence this process.

Additionally, the periodate oxidation reaction is exothermic, and the periodate has relatively low light stability. As a result, the reaction is typically performed in the dark at a temperature below 30 °C and at a pH between 3–7 [49].

Generally, the dialdehyde product properties depend on the processing parameters, such as periodate amount, temperature, and pH. Theoretically, the aldehyde content is affected by the improvement in periodate dosage. However, with the excessive molar ratio of periodate to the monomeric unit of polysaccharide, the aldehyde content will slightly increase due to the generation of hemiacetal, which is formed between an aldehyde group and a hydroxyl group at higher aldehyde content in the dialdehyde product [50]. For example, sodium alginate oxidation possessed an oxidation degree limit close to 50% due to the formation of stable inter-residue hemiacetals, which protect the hydroxyl groups from further oxidation [43,51]. As previously mentioned, the temperature affected the density of the dialdehyde product. Higher temperatures led to the deterioration of periodate ions, reducing the aldehyde density. In order to obtain the highest aldehyde density, the oxidation temperature must be no higher than 30 °C, as Kholiya et al. demonstrated, and they also confirmed that the aldehyde density of the dialdehyde–carboxymethyl agarose could be reduced from 81% to 64% as the temperature increase from 30 °C to 60 °C [52]. The aldehyde density also depends on the pH of the reaction media. In order to obtain a higher dialdehyde density in the oxidized polysaccharides’ chemical structure, the pH range must be between 3 and 7 [49]. The dialdehyde product will be subjected to acidic degradation at low pH (≤2.0), and at high pH (higher than 7), the aldehyde content and molecular weight will be reduced by the β-elimination reaction [49].

Moreover, due to the Cannizzaro reaction, some aldehyde groups can be further oxidized under alkaline conditions to carboxyl groups [53]. In addition to these factors, the type of the saccharide blocks can affect the generation rate of the dialdehyde groups. For example, the periodate oxidation for the guluronic residues of alginate was about 50% faster than the mannuronic residues [51]. Consequently, understanding the effect of periodate oxidation factors and the polysaccharides’ structural characterization is significant in forming high dialdehyde content with the appropriate structure.

When isolating derivatives of periodate oxidation products, caution is necessary to ensure that these products are not unintentionally modified in undesirable or unknown ways. One potential issue arises from the precipitation of free iodine, which can have harmful effects. This is because iodine has been shown to react with certain periodate oxidation products, leading to undesired alterations. Moreover, treating the common dialdehydes oxidation product with a strong base carries risks, as it can induce an internal Cannizzaro reaction. This reaction, exemplified by the oxidation of methyl P-L-rhamnopyranoside, can occur quantitatively within a short time frame when exposed to strong bases, like N sodium hydroxide, at elevated temperatures [54].

Furthermore, water can hydrolyze polysaccharides under certain conditions, especially in acidic or alkaline environments. This hydrolysis reaction cleaves glycosidic bonds in polysaccharides to form smaller oligo- or monosaccharides. While hydrolysis is not typically desired during oxidation reactions, it can occur as a side reaction if the reaction conditions are not carefully controlled [51,52].

In order to prevent undesirable reactions, like the precipitation of free iodine or the induction of internal Cannizzaro reactions when isolating derivatives of periodate oxidation products, several precautions can be taken. Firstly, it is crucial to completely remove the periodate excess from the reaction mixture before proceeding with the isolation. Purification steps, such as dialysis or chromatography, can effectively separate the desired oxidation products from unreacted periodate, thus preventing the formation of free iodine. Optimizing reaction conditions during periodate oxidation is also crucial to minimize the formation of unwanted by-products and side reactions. Controlling factors, such as pH, temperature, and reaction time, can promote the selective formation of desired oxidation products [54,55].

### 2.2. Functionalities and Characteristics of Polysaccharides Modified by the Addition of Carbonyl Groups

Polysaccharides featuring carbonyl functionalities exhibit unique characteristics and functionalities that make them valuable in various applications, particularly in biomedical, pharmaceutical, and industrial fields. Carbonyl groups (such as aldehydes and ketones) in polysaccharides can significantly alter their chemical reactivity, physical properties, and interaction with other molecules.

#### 2.2.1. Functionalities

##### Chemical Reactivity

- ***Cross-Linking Ability***

Introducing carbonyl groups into polysaccharides significantly enhances their cross-linking ability, forming stable networks with different applications across various fields. Polysaccharides that naturally contain hydroxyl groups can be subject to modification, allowing for the incorporation of carbonyl groups, specifically aldehydes or ketones, through oxidation reactions. The presence of resulting carbonyl groups in oxidized polysaccharides introduces new functionalities and opportunities for cross-linking through various chemical reactions, including Schiff base formation [56], Michael addition [57], and reductive amination [58].

- ***Oxidation–Reduction Reactions***

Carbonyl groups within polysaccharides participate in redox reactions, offering potential applications in sensors and responsive materials. These reactions enable the detection of analytes or environmental stimuli, making them valuable in sensor technologies. Polysaccharide-based materials can exhibit reversible changes in properties, such as conductivity, color, or mechanical strength in response to changes in temperature, pH, or specific chemical species [56].

- ***Bioactivity***

Polysaccharides modified with carbonyl groups have shown promising antimicrobial properties, making them valuable in combating microbial infections [56]. Carbonyl groups in these polysaccharides enable interaction with microbial cell membranes, leading to membrane disruption and cell death [57]. Additionally, carbonyl-containing polysaccharides can interfere with microbial metabolism, inhibiting vital cellular processes and impeding microbial growth and proliferation [58]. These antimicrobial properties are particularly beneficial in medical applications, where the prevention and treatment of infections are crucial [59]. By harnessing the antimicrobial activity of carbonyl-functionalized polysaccharides, novel antimicrobial agents, wound dressings, and medical device coatings can be developed to improve patient outcomes and reduce the spread of infectious diseases.

On the other hand, the introduction of carbonyl groups into polysaccharides significantly enhances their potential for drug-delivery applications [60]. Carbonyl functionalities provide reactive sites for forming Schiff bases or other chemical conjugates with therapeutic agents. This functionalization allows for precise control over drug loading and release kinetics, enabling targeted delivery to specific tissues or cells [60,61]. Moreover, the controlled release of drugs from these conjugates offers prolonged drug delivery, reducing the frequency of administration and improving patient compliance [62,63].

#### 2.2.2. Characteristics

##### Enhanced Hydrophilicity

Carbonyl-functionalized polysaccharides exhibit increased hydrophilicity, enhancing their water solubility and swelling capacity [64,65,66]. This property finds applications in hydrogels, wound dressings, and tissue engineering scaffolds [16]. In hydrogel formulations, the improved hydrophilicity facilitates efficient water absorption, enhancing gelation properties and mechanical stability [16]. Hydrogels derived from oxidized polysaccharides play a crucial role in wound care by promoting wound hydration and exudate absorption, which are imperative for optimal wound healing [67]. Additionally, in tissue engineering, their high hydrophilicity promotes cell adhesion, proliferation, and differentiation, facilitating the development of functional tissue constructs [67,68].

##### Mechanical Properties

Cross-linking via carbonyl functionalities enhances polysaccharide-based materials’ mechanical strength and elasticity, making them ideal for structural applications [69]. These covalent bonds reinforce the molecular network of polysaccharides, increasing resistance to deformation and improving tensile strength and toughness. Additionally, carbonyl-functionalized polysaccharides exhibit enhanced elasticity, allowing for reversible deformations without damage [69,70,71], making them suitable for several applications, like tissue engineering scaffolds, drug delivery, and in biomaterial implants, where mechanical integrity is crucial.

##### Biocompatibility and Biodegradability

Polysaccharides containing carbonyl groups maintain natural polysaccharides’ innate biocompatibility and biodegradability, making them highly suitable for medical and environmental applications requiring non-toxic and environmentally friendly materials [26,72]. In medical contexts, such as tissue engineering and drug delivery, these polysaccharides are well-tolerated by the body, minimizing the risk of adverse reactions or tissue rejection [73,74,75]. Furthermore, their biodegradability ensures safe metabolism and elimination, reducing long-term environmental impact. Similarly, carbonyl-functionalized polysaccharides support sustainable practices and pollution reduction in environmental applications, like biodegradable packaging and water treatment [76,77].

##### Controlled Gelation and Swelling

The degree of cross-linking and the density of carbonyl groups in polysaccharides can be finely tuned to regulate the material’s gelation behavior and swelling properties. This tunability is essential for tailoring materials with specific mechanical and rheological characteristics to meet diverse application requirements. By adjusting the extent of cross-linking, it is possible to modulate the gelation kinetics and the final mechanical strength of the resulting hydrogel or network [72]. Patenaude et al. developed injectable, covalently in situ-forming hydrogels based on poly(N-isopropyl acrylamide) by combining hydrazide-functionalized nucleophilic precursor polymers with electrophilic precursor polymers containing ketone (slow reacting) and aldehyde (fast reacting) functional groups. The results showed that increasing the ketone content in the precursor copolymers results in in situ-gellable hydrogels with better transparency and biocompatibility while maintaining equivalent mechanical properties and stimuli-responsiveness, albeit with a slight reduction in the speed of gel formation [78]. Similarly, varying the density of carbonyl groups influences the interactions between polymer chains, affecting the swelling capacity and responsiveness of the material to external stimuli, such as pH, temperature, or solvent composition [79,80]. This precise control over gelation and swelling properties enables the design of materials with tailored mechanical properties, stiffness, elasticity, and responsiveness, making them suitable for various applications, including drug delivery, tissue engineering, and controlled release systems.

### 2.3. Example of Polysaccharides Modified by Oxidation

#### 2.3.1. Alginate

**Figure 3:**
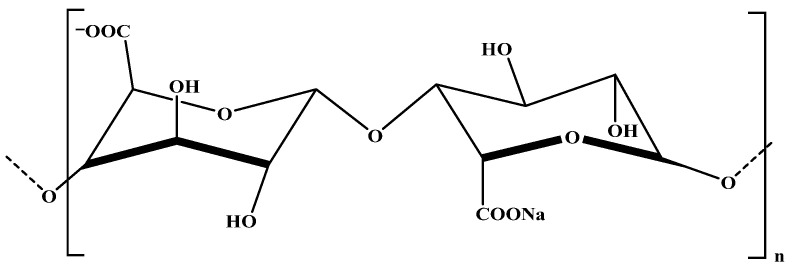
Chemical structure of the sodium alginate molecule.

Alginates are a significant class of natural polysaccharides extracted from marine brown algae, composed mainly of linear chains of 1,4-linked β-D-mannuronate (M) and 1,4-linked α-L-guluronate (G) residues and their sodium salts [81,82,83]. These two different repeating monomers could form regions with varying proportions of M residues (MM blocks), G residues (GG blocks), or alternating M and G structures (MG blocks) [84,85,86]. The source and species that produce the alginate may specify the M and G residues in the alginate chain as well as the physical properties of the polymers [84]. Thanks to its excellent biocompatibility [87], biodegradability and immunogenicity, non-toxicity, and functional groups [88], the alginate has been widely used in biomedical work, including as a drug carrier material [89], in tissue engineering [18], and for controlled-release purposes [90], as well as cell immobilization [91]. Figure 3 shows the chemical structure of the sodium alginate molecule.

Sodium alginate can be modified by the oxidation reaction using sodium periodate as an oxidizing agent in an aqueous solution. The oxidized alginate molecular weight decreased [50], its reactivity and biodegradability were improved, and the application fields increased [29,46]. Aldehyde groups can be generated in the ring-opening reaction by selective cleavage of carbon–carbon bonds in the uronic residues (C2–C3), decreasing alginate molecular weight [82,84]. The higher reactivity of the dialdehyde product occurs as a spontaneous interaction between the aldehyde groups and vicinal hydroxyl groups present on the unoxidized residues. The intramolecular and intermolecular hemiacetals can reduce the content of aldehyde groups formed [51]. Moreover, Painter observed that alginate possessed limited oxidation of close to 50% due to the formation of stable inter-residue hemiacetals [51].

The oxidation of alginate using periodate has been extensively studied. Gomez et al. oxidized sodium alginate with sodium periodate at room temperature for 24 h in the dark. This process caused a decrease in alginate stiffness by breaking the C2–C3 bond and cleaving the backbone simultaneously. As a result, the molecular weight decreased rapidly until the oxidation degree reached only 10 mol%, after which it remained constant. Also, they observed that gel was not formed in the presence of excess calcium cations until this oxidation degree was reached [83].

In their study, Ding et al. synthesized oxidized sodium alginate with varying sodium periodate concentrations (between 0.2:1, 0.4:1, 0.6:1, 0.8:1, and 1:1). The resulting samples were fractionated through graded ethanol precipitation, which yielded four oxidized sodium alginate fractions with a narrower molecular weight range. The researchers characterized the structure and properties of OSA samples and fractions using FTIR, AFM, viscosity measurements, and determination of aldehyde group content and then investigated the cross-linking performances of oxidized sodium alginate fractions on collagen fiber. Generally, they observed that the molecular weight of oxidized sodium alginate is a crucial factor in enhancing the properties of the cross-linked collagen fiber. The reduced molecular weight of OSA enhances its ability to infiltrate and interact within the complex structure of collagen fibers. This results in increased thermal stability and improved dispersion of collagen fibers [50].

A study by Balakrishnan et al. examined the oxidation of sodium alginate in both an aqueous solution and a 1:1 ethanol–water mixture. The aim was to compare the oxidation in these two different media to obtain a higher quantity of the oxidized product with a minimum amount of solvent in one reaction. The results showed that the oxidation kinetics and facile oxidation were similar in both mediums. Additionally, the cleavage of the dihydroxyl groups in the oxidation reaction occurred in both media, but it was more extensive in the ethanol–water medium. The product yield was also higher in this medium (50–60%) compared to the product medium in water (25–35%). Notably, oxidizing the alginate in a dispersion yielded larger quantities of the oxidized product with higher efficiency while consuming less solvent, making it an attractive approach for large-scale applications [82].

#### 2.3.2. Chitosan

**Figure 4:**
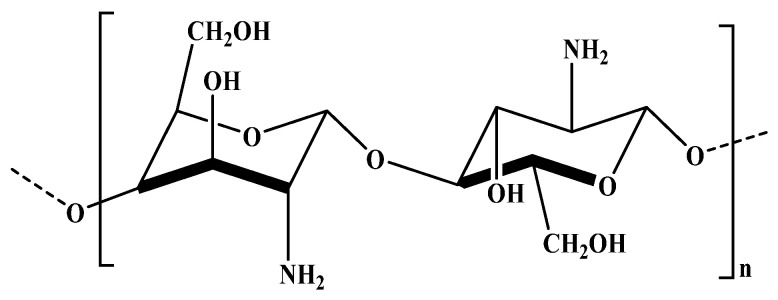
Schematization of the chemical structure of the chitosan molecule.

Chitosan is a linear, unbranched polysaccharide family produced through partial deacetylation of chitin found in crustacean shells [92,93,94]. It consists of two monomers, D-glucosamine and N-acetyl glucosamine, with varying amounts [93,95]. Chitosan has been distinguished by its unique properties, such as biocompatibility, biodegradability [93], non-toxicity, and antibacterial effects [92,94], and, hence, it is used in several areas, including the field of pharmaceutics, biomedical work [96], food, and in the fabrication of sensors and biosensors [94]. The structural unit of chitosan is characterized by the fraction of acetylated units (FA) since the glucosamine and N-acetylglucosamine residues are spontaneously distributed along the polymer backbone [94]. Figure 4 shows the schematization of the chemical structure of the sodium chitosan molecule.

The pH can easily affect the charge state of the glucosamine units’ primary amine (at the C-2 position). At a pH lower than 6.5 (in an acidic medium), the amino group in the chitosan backbone is protonated, and the chitosan can act as a water-soluble cationic polyelectrolyte. However, the chitosan loses its charge at a higher pH (>6.5) and becomes insoluble. This soluble–insoluble transition occurs in physiological conditions, enabling the biopolymer to form polyelectrolyte complexes with anionic polymers, such as alginates, xanthan, and hyaluronan [43,94,97].

Periodate oxidation of chitosan has been used relatively rarely. The periodate ion, IO_4_^−^, reacts with the D residues of chitosan to cleave the vicinal diols (carbon–carbon bond) by an oxidation reaction, which leads to the formation of a dialdehyde group. Moreover, it cleaves other 1,2-dioxygenated groups and 1,2-amino alcohols [96,97] and releases the ammonia. This ring-opening of pyranosidic residue may enhance the polymer’s flexibility and reduce its molecular weight [43].

Periodate oxidation of chitosan with different deacetylation degrees (FA = 0.05–0.65) and various molecular weights (Mw = 36,000–460,000) was studied by Bjørn et al. They found that the partial periodate oxidation of chitosans increased chain flexibility, as confirmed by a gradual decrease in persistence length, which serves as a characteristic length scale, indicating the distance over which two points on a polymer chain lose their correlation with each other (approximately 2 nm for the most oxidized samples) [98]. In the second study of the same research team, they investigated the reaction kinetics and stoichiometry, as well as the effect of the experimental conditions on the reaction product. Moreover, they focused on the role of FA and further evaluated the release of ammonia and the nitrogen/carbon (N/C) ratio measurements to establish the dialdehyde content or the oxidation degree [99]. The periodate oxidation reaction can increase the flexibility and solubility of the chitosan chain. Still, it may lead to depolymerization of the polymer, which could affect its ability to form polyelectrolyte complexes with polyanions. As a result, research on using periodate oxidation of chitosan to create new derivatives is limited, because this method often results in significant depolymerization [43].

#### 2.3.3. Pullulan

**Figure 5:**
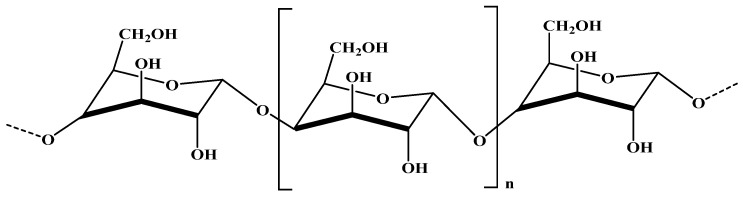
Schematic representation of the chemical structure of the pullulan molecule.

Pullulan is a natural exopolysaccharide that is produced in a fermentation medium by a yeast-like fungus called Aureobasidium pullulans [100]. It is a nonionic and linear polymer comprising maltotriose units joined through α (1→4) glycosidic bond. Consecutive maltotriose units are attached by α (1→6) glycosidic linkages [101,102]. This polymer is highly biodegradable, non-toxic, thermally stable, and has properties that can be modified. Therefore, it has become an ideal polysaccharide for various applications, including biomedical purposes, food, cosmetics, and tissue engineering [102,103,104,105]. Pullulan is a type of polysaccharide that has unique properties. Its bonds alternate between α (1→4) and α (1→6), which give it flexibility and solubility. Pullulan decomposes at high temperatures ranging from 250 °C to 280 °C. It is highly soluble in water but insoluble in organic solvents. It can only be diluted in an alkali solution. Pullulan’s aqueous solutions are more stable and have relatively low viscosity compared to other polysaccharides, like alginate [106,107]. Figure 5 represents a schematic representation of the chemical structure of the pullulan molecule.

Pullulan can be activated to obtain unique derivatives with modified structures and properties [108]. In recent years, the periodate oxidation of pullulan has been well-researched in many studies, especially as a bio cross-linker with the amino groups for developing hydrogels for biomedical applications [101,103,105,107]. This modification was carried out to cleave the vicinal diols of the maltotriose unit at the C2 and C3 carbon atoms, resulting in a reactive aldehyde group along the polysaccharide backbone [104,108]. The aldehyde groups on the modified pullulan can be cross-linked with the free amino groups on the proteins to create the Schiff base structures and form a cross-linked network [104]. Since 1993, Bruneel et al. have applied periodate oxidation to modify the polysaccharides. This study focused on examining the activated products’ structure and characterization. The researchers discovered that pullulan’s oxidation produces various dialdehyde structures. Pullulan’s structure comprises three distinct anhydroglucoside moieties in the repeating unit. Only 67% of the aldehyde content resulted in the complete oxidation of periodate-oxidized pullulan due to the formation of a stable six-membered hemiacetal [100]. Zhang et al. obtained gelatin-based hydrogels using a cross-linker prepared from pullulan that had been oxidized with periodate to form dialdehyde groups. They discovered that the pH of the aqueous phase had an impact on the aldehyde content. The study revealed that the aldehyde content increased when the pH was increased from 2 to 4, reached 5.48 mmol/g, and then decreased when the pH exceeded 4 [108].

Liu et al. synthesized a range of oxidized pullulan derivatives with varying molecular weights and aldehyde contents using periodate oxidation of pullulan. These derivatives were then incorporated into edible gelatin films, facilitating chemical cross-linking between the protein chains. As a result, the addition of oxidized pullulan enhanced the gelatin films’ water vapor barrier properties and thermal characteristics [102]. Furthermore, Selvakumar and colleagues successfully oxidized pullulan with sodium periodate to produce an aldehyde derivative, utilized as a bio cross-linker for collagen stabilization [104].

#### 2.3.4. Carboxymethylcellulose

**Figure 6:**
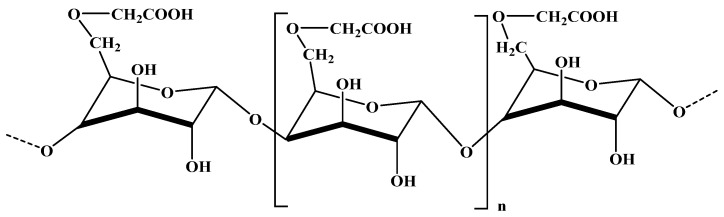
Schematization of the chemical structure of the carboxymethyl cellulose molecule.

Carboxymethylcellulose is a significant cellulose derivative that is produced under controlled conditions through the etherification reaction of alkaline cellulose with sodium monochloroacetate [109,110,111]. This water-soluble cellulose ether is available in various degrees of substitution [109] and is an anionic linear polymer that finds wide use in numerous industrial areas, such as food, biomedical applications, pharmaceuticals, cosmetics, mineral processing, paper-making, and tissue engineering. Its non-toxicity, biodegradability, and biocompatibility make it a popular choice in these industries [110,111,112,113,114]. Figure 6 presents the schematization of the chemical structure of the carboxymethyl cellulose molecule.

Li et al. investigated the hydroxyl groups of carboxymethyl cellulose conversion into dialdehyde derivatives in an acidic medium as a function of pH, temperature, reaction time, and periodate concentration. They found that the stoichiometric ratio of NaIO_4_ to carboxymethyl cellulose and the pH of the aqueous medium was substantially responsible for the aldehyde content and yield of the oxidized polysaccharide. However, the results confirmed a relation between the degradation and periodate oxidization [109].

In another study, Zhang et al. synthesized dialdehyde carboxymethyl cellulose by the periodate oxidation reaction in acidic conditions. The production of dialdehyde carboxymethyl cellulose was explored based on an optimization experiment using different periodate concentrations, temperatures, and pH mediums as variables. Their results showed that the effect of these three factors on the dialdehyde group production was in the following order: pH medium > temperature > periodate concentration. The optimum conditions in which the dialdehyde yield reached 79.04% involved a pH of 3.0, a reaction temperature of 30 °C, and a weight ratio between NaIO_4_ and carboxymethyl cellulose of 1:1 for a reaction time of 4 h [115]. Using two methods, Kulikowska et al. oxidized the carboxymethyl cellulose to produce aldehyde groups. In the first method, the conversion of carboxymethyl cellulose to its dialdehyde derivatives took place by using sodium periodate as the oxidizing agent. In the second one, they used the hydrogen peroxide oxidizing agent for the oxidation reaction in the presence of iron tetrasulfophthalocyanine as a catalyst. In both methods, the oxidation is carried out under different conditions (pH, temperature, and reaction time). The results showed that the aldehyde groups were introduced using both methods, and the results confirmed that the first method was the most efficient and gave results similar to those from the theoretical calculations. In this study, the oxidation reaction’s optimum conditions were pH = 7 for 24 h at 22 °C with 1 g of carboxymethyl cellulose using 1.51 g of sodium periodate [111].

Carboxymethyl cellulose has been extensively researched for its oxidation properties when introducing dialdehyde groups. This has proven to be particularly useful in developing biodegradable and biocompatible hydrogels for drug delivery. Dialdehyde carboxymethyl cellulose has proven to be an effective cross-linker with the amino groups in the protein chain during the synthesis process [116,117,118].

#### 2.3.5. Pectin

**Figure 7:**
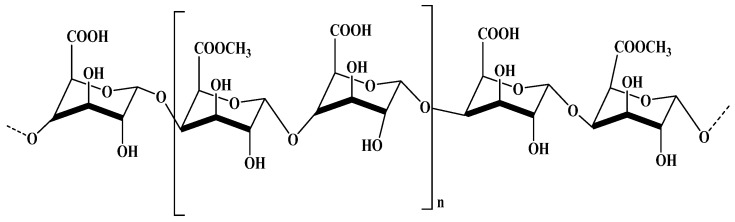
Chemical structure of pectin molecule.

Pectin is a natural polysaccharide present in the cell walls of plants, as well as in apple pulp, citrus fruits, and beetroot. It is composed of a poly 1,4-galacturonic acid unit and is classified as an anionic polysaccharide. Pectin is widely used in the biomedical field due to its biocompatibility, non-toxicity, hydrophilicity, and biodegradability. It is commonly used as a drug delivery system in the colon because it can be degraded in the colon. When exposed to acidic conditions, pectin molecules can interact with each other to form a gel-like structure. The carboxyl groups on galacturonic acid residues are less dissociated in acidic environments, reducing the electrostatic repulsion between pectin chains resulting in the characteristic texture of a gel [119]. Pectin hydrogels are highly compatible with living cells and have excellent gelling properties, making them ideal for drug delivery and other biomedical applications [120,121,122,123,124]. Figure 7 presents the chemical structure of the pectin molecule.

Modifying pectin can provide numerous opportunities to transform this polysaccharide into an interesting product. Periodate oxidation is one important modification method for pectin, by which the vicinal diols that are present at the C2–C3 carbons of the D-glucopyranose unit from pectin are cleaved, and the pectin backbone tends to degrade, leading to a reduction in molecular weight and the formation of carbonyl groups along its chain [124,125]. Gupta et al. oxidized methyl citrus pectin using periodic acid in an aqueous and ethanol–water mixture solution. In order to produce a higher quantity of dialdehyde groups along the polysaccharide backbone, they investigate the effect of the reaction conditions on the oxidation process, such as reaction time, temperature, pH medium, and periodic acid concentration in both mediums (water or water–ethanol mixture). Their results showed that the aldehyde content increased when the reaction time, periodate concentration, and temperature of the reaction medium were raised at an acidic pH in both mediums. However, the reduced intrinsic viscosity indicated that the degradation occurred simultaneously with the polysaccharide oxidation. The researchers found that when the amount of ethanol is greater than the amount of water in a water–ethanol mixture, the aldehyde content increases, which is opposite to what they initially suggested. The initial suggestion was based on the idea that polysaccharides are not soluble in ethanol. However, the researchers explained this contradiction by pointing out that the formation of hemiacetals is hindered in water–ethanol systems due to the presence of steric hindrances, as previously discussed in Gupta et al.’s study [120]. The researchers obtained a cross-linking hydrogel based on biopolymers, and they demonstrated the formation of Schiff bases between aldehyde groups from oxidized pectin and amino groups of gelatin under various reaction conditions [121].

Munarin et al. functionalized pectin by treating it with sodium periodate and coupling it with an oligopeptide containing arginine–glycine–aspartic acid (RGD) motifs, and then the product obtained was subjected to a comprehensive characterization process, including an assessment of the degree of esterification, intrinsic viscosity, molecular weight, rheological properties, biological performance, and structural attributes. This research demonstrated the effectiveness of partial oxidation and RGD grafting in regulating the degradation and cytocompatibility of pectin microspheres. Specifically, RGD–pectin microspheres exhibited the highest level of cytocompatibility, while oxidized pectin microspheres showed accelerated dissolution, indicating the potential for quicker in vivo cell release. This study has established the potential of these biomaterials as injectable cell delivery systems with promising applications in tissue engineering and the broader field of regenerative medicine [126]. The Table 1 shows some examples of oxidized polysaccharide properties.

## 3. Hydrogels

Hydrogels are a unique class of cross-linked natural or synthetic polymer networks that can absorb and retain a significant quantity of aqueous solutions and biological fluids [127,128,129]. This holding water capacity arises due to the hydrophilic polymer network, which might be produced via in situ physical or chemical cross-linking [56,127]. Usually, the physical networks have been constructed by non-covalent interactions, such as hydrogen bonding, ionic interactions (e.g., electrostatic interactions in polyelectrolyte complexes), metal coordination (e.g., the formation of hydrogels using metal-coordinated ligands), hydrophobic interaction (e.g., aggregation of hydrophobic segments in proteins), and host–guest interaction (e.g., cyclodextrin inclusion complexes). However, chemical cross-linking can be generated using different methods, such as polymerization cross-linking (e.g., acrylamide-based monomers cross-linked via radical polymerization to form polyacrylamide hydrogels), ionic cross-linking (e.g., alginate hydrogels cross-linked with divalent cations, such as calcium ions), using a chemical cross-linking agent (chemical cross-linking agents, such as glutaraldehyde or genipin, can be added to react with functional groups on polymer chains.), Michael addition (e.g., formation of hydrogels through the Michael addition of thiol-containing polymers [130]), enzymatic cross-linking (e.g., cross-linking of gelatin using transglutaminase enzymes to form gelatin hydrogels [131]), metal coordination cross-linking (coordination bonds form between polymer chains and transition metal ions), and photochemical cross-linking (cross-linking induced by exposure to UV light, e.g., photopolymerization of acrylate-based monomers to form photo-cross-linked hydrogels) [56].

Hydrogels can be developed via cross-linking a single or mixture of water-soluble polysaccharides, encompassing a large range of chemical and physical properties. On the other hand, hydrogels might be formulated in different physical forms, such as slabs (sheets), microparticles, nanoparticles, coatings, and films [127]. In recent years, hydrogels have gained significant interest, particularly in biomedical and pharmaceutical applications. They have been used to facilitate the controlled release of drugs, especially proteins, and for encapsulating living cells. Furthermore, hydrogels were used in other biomedical applications, such as in contact lenses, artificial corneas, wound dressing, and coating for sutures [127,128].

### 3.1. Synthesis of Hydrogels

Hydrogels are flexible three-dimensional network polymers that have cross-linking as their fundamental characteristic. The density of cross-linking significantly impacts the performance of hydrogels. This density can be achieved through various techniques, such as physical cross-linking, chemical cross-linking, grafting polymerization, and radiation cross-linking. These modifications can enhance hydrogels’ viscoelasticity and mechanical characteristics for use in different biomedical and pharmaceutical industries [56,132].

#### 3.1.1. Chemical Cross-Linking

**Figure 8:**
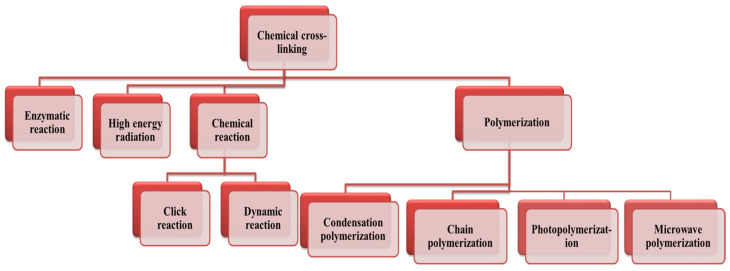
Diagram representing different chemical methods of hydrogel fabrication.

Chemically cross-linked hydrogels are formed when covalent bonds are created between the chains of one or several polymers. These cross-links are obtained through various methods, such as polymerization with multifunctional monomers, the application of high-energy radiation, chemical reactions of complementary or pendant groups, or reactions with cross-linking agents. Enzymes can also be used as cross-linking agents [129,132]. Figure 8 shows different methods to synthesize chemically cross-linked hydrogels.

Polymerization with a multifunctional monomer can be achieved using step or condensation polymerization and free radical or chain polymerization [133]. The reactions of these polymerization techniques can be carried out in solution, bulk, or suspension. The polymerization is started by adding a small amount of an initiator after combining the monofunctional and multifunctional monomers [134,135]. Among the various polymerization techniques used to develop hydrogels, photopolymerization and photo-cross-linking are among the most versatile methods to obtain hydrogels due to several benefits: (1) they use chemical reactions that are insensitive to water, making them ideal for aqueous environments; (2) they are generally very rapid processes, allowing for the creation of self-supporting hydrogels in seconds or minutes; (3) they provide precise spatial and temporal control over the cross-linking process, which is especially useful in stereolithography and 3D-(bio)printing techniques; (4) they exhibit low cytotoxicity when conducted under optimal conditions, thereby having minimal adverse effects on cell survival and proliferation [136,137]. On the other hand, microwave-assisted polymerization has emerged as an effective technique for preparing hydrogels. The high temperatures generated by microwave heating accelerate reactions compared to conventional thermal methods. This approach offers a potentially faster, more efficient, and selective method for the thermal treatment of biomass, facilitating the rapid conversion of monomer solutions into gels or solids [138,139,140].

Cross-linking can occur through various mechanisms, such as free radical or oxidizing reactions, which are assisted by an increase in temperature and the presence of air. Alternatively, UV light or other radiation sources can also promote cross-linking. Another mechanism involves a chemical reaction, for instance, the condensation reaction of an alcohol or an amine with a carboxylic acid. Cross-linking of polymers creates a three-dimensional network structure when chemical bonds or bridges form between the polymer chains. This process improves the polymer’s mechanical properties and stability. Cross-links can be formed through chemical reactions initiated by heat, pressure, pH changes, or irradiation [141].

A wide range of initiators, such as azo compounds, peroxides, and redox initiators, can be used to initiate polymerization. The main benefit of chemical initiation is the production of comparatively pure, residue-free hydrogels [133]. High energy and electromagnetic irradiation yields, especially gamma and electron beams, can be used for chemically cross-linking the water-soluble monomer or polymer by generating free radicals on polymer chain ends without the addition of a cross-linker [129,132]. This high-energy irradiation can convert the vinyl groups of water-soluble polymers into hydrogels. The resulting radicals combine to form bonds between polymer backbones, resulting in a cross-linking polymer network [128,129,132].

**Figure 9:**
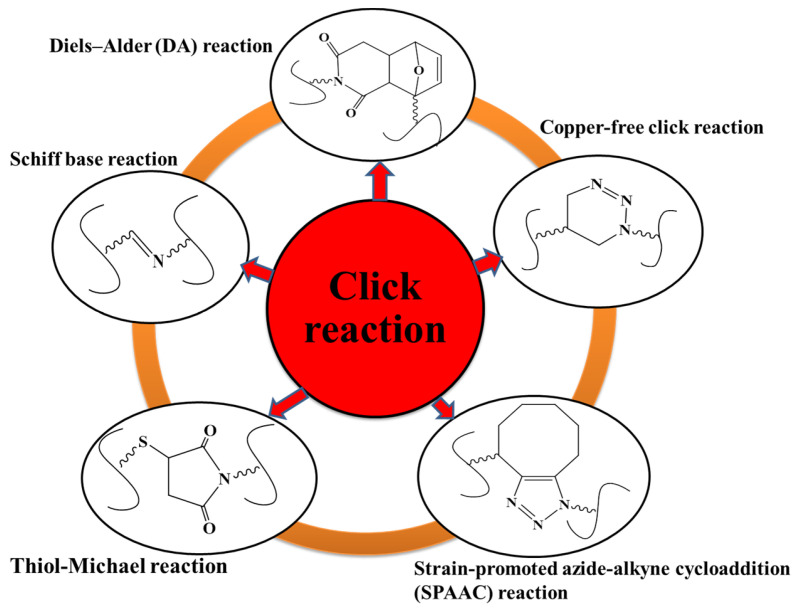
Click chemistry reactions employed to cross-link hydrogels.

Furthermore, chemically cross-linked hydrogels can be developed by click chemistry and dynamic reaction, especially cross-linking reactions by Schiff base formation for the water-soluble polymers [56,131]. The click reactions are considered to be faster, more versatile, more regiospecific, and more efficient than the Schiff base reactions, leaving no by-products [132,134]. Hydrogels can be obtained using click reactions, including azide–alkyne cycloadditions [132], Diels–Alder, thiol–ene, and oxime reactions. In the case of cross-linking with Schiff base formation, the polymers must have functional groups, such as alcohol, amine, or hydrazide, which can react with aldehydes to form the polymer cross-linking network [133]. Figure 9 shows the click chemistry reactions employed to cross-link hydrogels.

**Figure 10:**
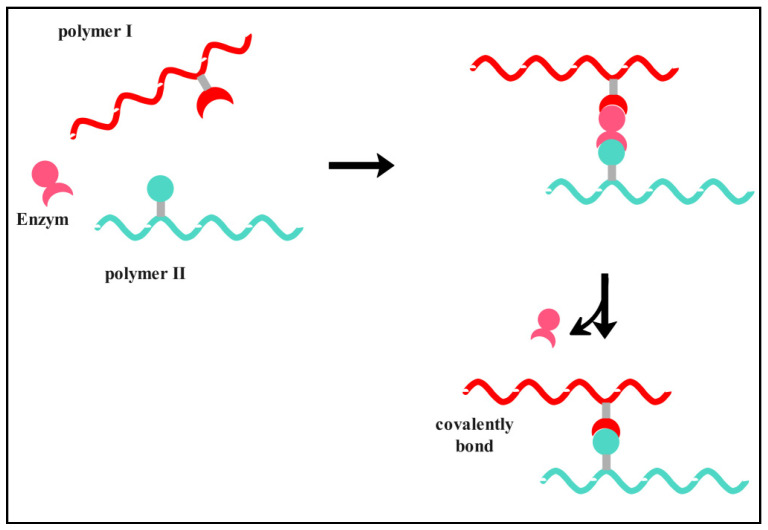
Schematic of enzymatic cross-linking as a catalyst that assembles two functional groups of different polymer chains. Adapted from [146].

There is another mechanism using enzymes as cross-linking agents. This mechanism is considered attractive due to the enzyme specificity and the mild reaction conditions required for cross-linking. The cross-linking takes place in two ways: direct enzymatic catalysis of cross-link formation, as shown in Figure 10, or indirectly using an enzymatic production of a cross-linking agent, such as H_2_O_2_, which in turn is able to oxidize reactive structures with subsequent cross-link formation [142,143,144]. Jus et al. investigated enzymatic protein cross-linking as an alternative to traditional methods, which often rely on toxic chemicals. They explored the use of tyrosinases from various sources and laccases to cross-link gelatine and gelatine hydrolysates. Through spectroscopic analyses and oxygen consumption measurements, enzymatic oxidation of tyrosine residues was confirmed. Dimerization of a model substrate was observed using chromatographic techniques. Enzymatic cross-linking significantly increased the molecular weight, leading to material precipitation [143].

Additionally, the presence of phenolic molecules enhanced the cross-linking effect. This study highlights the potential of enzymatic approaches for protein cross-linking, offering a safer and more eco-friendly alternative to traditional chemical methods [143]. In another study, Rossi et al. determined the effectiveness of whey protein/pectin edible films by adding transglutaminase as a water barrier coating for fried and baked foods. They applied these hydrocolloidal films to doughnuts, French fries, and taralli biscuits to assess their impact on moisture loss, oil absorption, and texture properties. The results demonstrated significant reductions in moisture loss and oil content in coated fried foods compared to uncoated samples. Sensory evaluation tests revealed no texture differences between coated and uncoated products. Furthermore, the edible films effectively prevented moisture absorption by taralli biscuits during storage, maintaining their desired texture. Overall, the study highlights the potential of whey protein/pectin edible films to enhance fried and baked foods’ quality and shelf life [144].

Furthermore, Battisti et al. developed a novel packaging approach by coating raw paper sheets with biopolymeric solutions comprising gelatin cross-linked with transglutaminase enzyme, glycerol, and citric acid. Despite there being no enhancement in mechanical properties, the coating exhibited effective film formation, as confirmed by various analyses. It maintained the paper’s optical properties while significantly reducing water vapor permeability. When used for beef packaging, the coated papers demonstrated several benefits: lower microbial populations after storage, enhanced stability against lipid oxidation, preserved red color, and lower pH values compared to uncoated paper. Moreover, they effectively prevented moisture loss from the beef, indicating their promising role in food preservation and quality maintenance [145].

#### 3.1.2. Physical Cross-Linking

Physical hydrogels have recently received significant attention in pharmaceutical, biomedical, and food applications due to their relatively easy fabrication protocol, avoiding cross-linking agents [127,129,147]. Physical hydrogels often have a reversible structure with continuous, dynamic, non-covalent bonds. The physical hydrogels form through reversible intra/intermolecular bonds, and their association–dissociation dynamics depend on factors, such as pH, temperature, and ionic strength. Contrary to the chemical hydrogels, their cross-links are composed of non-covalent bonds of lower energy [147,148,149]. These bonds give the hydrogels lower mechanical properties than chemical bonds [148,149]. In order to synthesize physical hydrogels, several methods have been investigated, such as intra- and intermolecular bonds (hydrogen bonds, hydrophobic association, van der Walls interactions), ionic cross-linking, stereo-complexation, and electrostatic interactions. Physical hydrogels made from proteins can be created by changing the pH or exposing the protein to a temperature above its denaturation point [148].

Physical hydrogels created by ionic cross-linking are three-dimensional polymeric networks cross-linked by ion interactions under mild conditions (at room temperature and physiological pH) [128,150,151,152]. Ionic cross-linking can also occur by the interaction between some di or multivalent metal ions, such as Ca^2+^, Fe^3+^, Cu^2+^, and Zn^2+^, and polymer functional groups, for example (carboxylic). These interactions lead to ionically cross-linked hydrogels [147,151,152], such as alginate hydrogels [56,129,152,153]. It has been suggested that physically cross-linked hydrogels can be obtained by forming hydrogen bonds. When a hydrogen atom bonds with an atom that has a higher electronegativity, like oxygen, nitrogen, or fluorine, hydrogen bonds occur. In this case, the hydrogen atom takes a partial positive charge, while a negative charge exists on the electronegative atom, leading to electrostatic interactions between them [148,154]. Hydrogen bonds act as physical cross-links (inter- or intramolecular) during hydrogel formation [155,156,157]. The primary interaction type for the formation of physical hydrogels based on natural polymers is hydrogen bonding, as they contain several functional groups in their chains, such as hydroxyl, amino, and carboxylic acid groups [151]. Stereo-complexation hydrogels were obtained by synergistic interaction between polymer chains or molecules containing the same chemical composition but opposite stereochemistry [149].

Well-known examples of stereo-complexation hydrogels are those produced from poly(lactide) copolymer. Poly(lactide) is a biodegradable polymer derived from renewable resources, often used in biomedical and pharmaceutical applications due to its biocompatibility and tunable properties. When poly(lactide) copolymers with different stereoisomeric structures are combined, such as poly(l-lactide) and poly(d-lactide), they can form stereo-complexation hydrogels [158,159]. These hydrogels have a significant advantage, which is the simplicity of preparation (hydrogels can be obtained by mixing copolymers dissolved in aqueous solution) [149]. Physical hydrogels could also be formed through electrostatic interaction between oppositely charged polyelectrolytes in suitable reaction conditions without chemical cross-linkers [158,159]. Mixing the negative charge of polyanions and the positive charge of polycations during the formation of the polyelectrolyte complexes leads to strong and rapid interaction processes, resulting in phase separation in the solution [160]. These hydrogels had several advantages, such as the absence of toxic chemical cross-linker agents, the rapidity and simplicity of the reaction under mild conditions, and the combination of the desired properties of two or more components [160].

Physical hydrogels created via hydrophobic association are generally formed by the aggregation and association of the hydrophobic groups of amphiphilic polymers generated by two methods, namely copolymerization of vinyl monomers, which contain double bonds with hydrophobic monomers, or by the introduction of a lower quantity of hydrophobic groups using a chemical reaction [160]. Under entropy, the hydrophobic groups are associated and aggregated, leading to the generation of the three-dimensional framework in aqueous medium. Physical cross-linking can be achieved by crystallization. The underlying principle of this method is that the gelatin or the polysaccharides are renaturated to the triple helical conformation in the case of gelatin or double helical conformation in the case of polysaccharides during the sol–gel reversible hydrogel formation, which induces the nucleation and growth of crystallites [150,160]. The helices aggregate and result in a junction point, which is affected by the temperature. The helices have a random coil shape at high temperatures. With the reduction in the temperature, they start to generate double helices and aggregates that act as knots (nodes), forming the hydrogels’ physical junctions (cross-linking points) [161].

## 4. Protein

Proteins are complex biological macromolecules made up of chains of amino acids. They are fundamental components of all living organisms and play crucial roles in various biological processes. Proteins are involved in structural support, enzymatic reactions, immune responses, cell signaling, transport of molecules, and many other functions within cells and organisms [162]. Proteins, as macromolecules, are integral components of cells, constituting more than 50% of the dry weight and surpassing all other biomolecules in abundance. They are noteworthy for their pivotal role in catalyzing and regulating biochemical reactions within biological systems. Furthermore, it is imperative to recognize and appreciate the essential contributions of other components of living systems, such as nucleic acids, lipids, and carbohydrates, which collectively and indispensably participate in the functions and processes of life [163].

The structure of a protein is hierarchical, with primary, secondary, tertiary, and sometimes quaternary levels of organization. The primary structure refers to the linear sequence of amino acids in the protein chain. The secondary structure involves folding the polypeptide chain into alpha-helices, beta-sheets, or other structural motifs stabilized by hydrogen bonds. The tertiary structure is the overall three-dimensional folding of the protein chain, driven by interactions between amino acid side chains and the surrounding environment. The quaternary structure, present in proteins with multiple subunits, refers to the arrangement of these subunits in the protein complex [164].

### 4.1. The Shape and Structure of Proteins

From a chemical standpoint, proteins represent the pinnacle of structural complexity and functional sophistication among known molecules. This complexity is not surprising, considering that each protein’s structure and chemistry have been honed over billions of years of evolutionary history. Even experts are often astounded by the remarkable versatility of proteins [165].

The shape of a protein is dictated by its sequence of amino acids, which are synthesized as linear chains of amino acids, forming a polyamide (polypeptide) structure. However, they adopt intricate three-dimensional shapes to fulfill their functions. While approximately 300 amino acids are found across various animal, plant, and microbial systems, only 20 amino acids are encoded by DNA for incorporation into proteins. Many proteins contain modified amino acids and associated components known as prosthetic groups [162,165].

Various chemical techniques are employed to isolate and characterize proteins based on criteria, such as mass, charge, and three-dimensional structure. Proteomics, an emerging field, focuses on studying the complete spectrum of protein expression within a cell or organism, including alterations in protein expression in response to certain factors, such as growth, hormones, stress, and aging [162].

### 4.2. Proteins-Based Hydrogels

**Figure 11:**
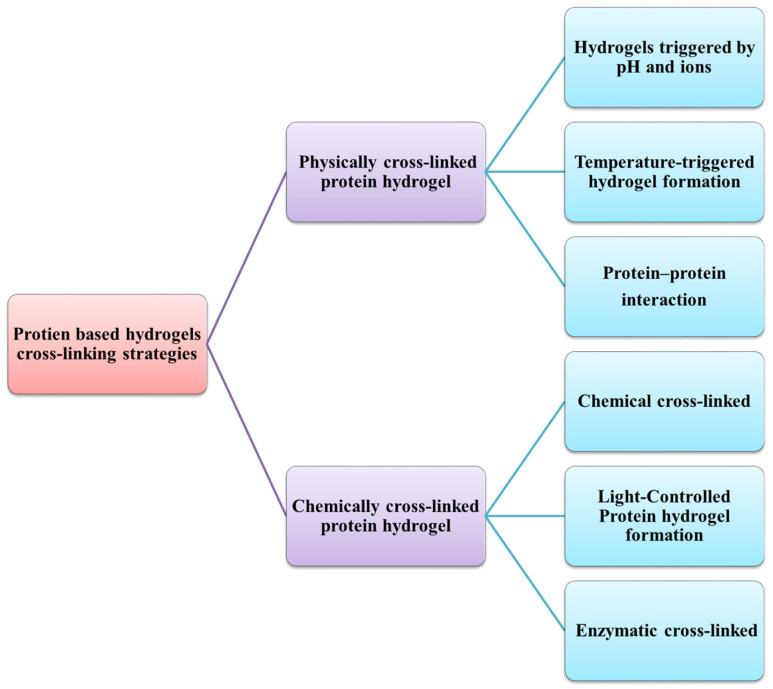
Protein-based hydrogels fabrication methods.

Protein-based hydrogels are highly attractive for various medical applications due to their biocompatibility, biodegradability, and ability to undergo chemical and biological modifications. Recent progress in protein engineering, synthetic biology, and materials science has made it possible to precisely adjust protein sequences, hydrogel structures, and mechanical properties, enabling various biomedical applications for protein hydrogels [148]. With their reactive side chains, proteins interact with other protein or non-protein molecules, such as polysaccharides or synthetic polymers, to generate cross-linked protein polymers, like hydrogels. These cross-linked polymers have the potential to replace toxic, non-degradable synthetic polymers [166]. Proteins can serve as the basis for hydrogel formation through several physically cross-linked mechanisms or chemical cross-linking (Figure 11). Physical cross-linking involves non-covalent or weak covalent bonds that are typically temporary or reversible using many strategies, such as changing the pH, ionic strength, or temperature [148,166]. On the other hand, chemical cross-linking can occur either through the use of multifunctional small-molecule cross-linkers or by incorporating specialized functional groups into proteins for covalent cross-linking that form relatively stronger bonds, leading to more stable cross-linked protein networks [148].

Protein hydrogels, such as silk and collagen-based hydrogels, are extensively explored for their biocompatibility and utility in tissue engineering. They serve as promising scaffolds for cell growth and tissue regeneration. The quality of naturally isolated proteins from different sources can vary significantly, leading to inconsistencies in the material properties of the resulting hydrogels. Furthermore, the inherent properties of natural proteins often restrict the potential characteristics that hydrogels can achieve, thereby limiting their versatility in various biomedical applications. Despite proteins’ crucial roles in bodily functions, hydrogels based on proteins face multiple challenges that must be addressed. These hydrogels typically possess low mechanical strength unless they are covalently cross-linked, presenting complex challenges for regenerating robust tissues, like bone, that require careful consideration and innovative solutions

Moreover, many lack growth factor-specific binding sites, hindering their ability to sequester these proteins, which are crucial for tissue regeneration and cell fate modulation without adverse effects, such as tumor growth [167,168,169]. In response to these challenges, scientists have developed various approaches to enhance the mechanical characteristics of protein-based hydrogels. These strategies involve physically or chemically modifying the hydrogels to improve their performance and functionality.

### 4.3. Strategies for Reinforcing the Gel Network

Enhancing the mechanical properties of protein-based hydrogels is crucial for expanding their applications across various fields. One effective strategy is chemical cross-linking, which involves using multi-functional cross-linkers [166,170] or incorporating specialized functional groups, such as acrylates, methacrylates, thiols, and amines, to facilitate covalent bonding within the hydrogel network [171,172]. Enzymatic cross-linking, using enzymes, like transglutaminase, can also strengthen the network by catalyzing the formation of covalent bonds between protein molecules [173]. Photo-cross-linking, which involves incorporating photoresponsive groups that form covalent bonds upon light activation, allows for precise control over the cross-linking process [174].

Blending protein hydrogels with other natural or synthetic polymers can improve their mechanical strength, elasticity, and durability. Incorporating nanomaterials, such as nanofibers and nanoparticles, into the hydrogel matrix further reinforces the hydrogel structure [175]. Physical cross-linking methods, including non-covalent interactions, like hydrogen bonding, ionic interactions, and hydrophobic interactions, as well as freeze–thaw cycling, can also enhance the stability and interlinking of the hydrogel network [166,176,177].

Modifying the hydrogels to be responsive to pH and ionic changes can enhance their functionality and mechanical resilience [158]. Additionally, recombinant protein engineering offers another approach, where proteins are genetically designed with specific sequences that include cross-linking sites or functional domains, improving mechanical properties. Fusion proteins, which combine functional domains from different proteins, can further enhance binding affinity and mechanical strength. These diverse strategies collectively aim to produce robust, versatile protein-based hydrogels suitable for various biomedical and industrial applications.

## 5. Cross-Linking with Carbonyl Derivatives of Polysaccharides—*Schiff Base Linkages*

Schiff base derivatives are a type of compound that was first discovered in 1864 by the German chemist and Nobel Prize winner Hugo Schiff [178,179]. These compounds are formed through a reaction between amino and carbonyl groups or benzoic aldehydes; they can be considered a sub-class of imines. The reaction between a carbon and nitrogen atom forms a double bond, creating an imine or azomethine functional group (–C=N–) [180]. As per the recommendation of the International Union of Pure and Applied Chemistry (IUPAC), Schiff bases are defined as chemical compounds (imines) that have a hydrocarbyl group at the nitrogen atom R2C = NR′ (where R′ is not equal to H). Many consider Schiff bases to be identical to azomethines [181]. The formation of Schiff bases can occur under mild conditions, producing water as the only byproduct [182]. These linkages can create imines, hydrazones, and oximes cross-links, which have numerous applications in various fields, including pharmaceuticals, biological studies, inorganic chemistry, and medicine. Scientists have also been exploring using novel heterocyclic/aryl Schiff bases to develop new environmentally friendly technologies due to their simplicity, versatility, the non-toxicity of the reagents and products, and their reversibility [179,180].

Schiff bases are a significant class of compounds due to their simple preparation using inexpensive starting materials, such as aldehydes/ketones and primary amines. It is easy to link biologically active scaffolds with simple azomethine linkage to create diverse molecular hybrids with varying biological properties. Schiff bases are versatile ligands for coordinating metal ions of various oxidation states and coordination numbers. The structure of Schiff base ligands can be easily tailored to coordinate different metal ions [183].

**Figure 12:**
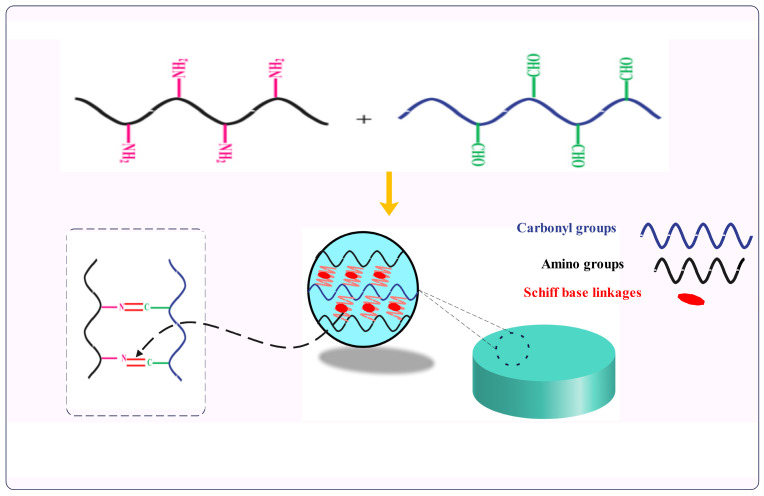
Schematic representation of the formation of Schiff base hydrogels.

Hydrogels are formed by Schiff base cross-linking, which involves mixing aldehyde/ketones groups with the amines, hydrazides, and aminooxy groups of biopolymers to produce imines, hydrazones, and oximes cross-links, respectively. These cross-links act as the junctions of hydrogels [16]. It has been observed that the hydrazone and oxime cross-links are more stable than imine linkages, which are a condensation product of aldehyde/ketone groups with α-effect nucleophiles containing amino groups next to nitrogen and oxygen atoms, respectively [16,181,182,183]. Additionally, the benzoic Schiff base hydrogels were fabricated by the linkage of amine, hydrazides, and aminooxy’s with benzaldehyde groups, resulting in benzoic imines, benzoic hydrazides, and benzoic oximes, respectively, to improve the carbon–nitrogen double bond (–C=N–) [16,184]. Figure 12 illustrates how Schiff base hydrogels are formed through a schematic representation. The polymers must be functionalized by introducing the aldehyde or nucleophilic groups to prepare hydrogels based on imine, hydrazone, or oxime cross-linking. Generally, the primary amino groups can be found in many native polymers; however, chemical modification is necessary to create the carbonyl groups in the other reagent [182]. There are two methods to functionalize the polysaccharides: by oxidizing vicinal hydroxyl groups of various polysaccharides using the sodium meta periodate or using the molecules containing aldehyde groups by carbodiimide chemistry [16].

The imine bond (or Schiff base) is a reversible dynamic covalent bond formed by the nucleophilic attack of amine derivatives to carbonyl groups (aldehyde/ketone) under physiological conditions [185,186]. The imine reaction involves the elimination of H_2_O molecules when the C=N bond is formed intra- or intermolecularly through the reaction of two molecules containing amino and carbonyl groups.

The rate of this reaction is accelerated by acid catalysis [185,187]. The imine bond is reversible, meaning that the reaction can take place in two directions. In the forward direction, water molecules are removed either physically using the Dean–Stark apparatus under azeotropic conditions or chemically by adding a drying agent to the reaction mixture, such as molecular sieves or MgSO_4_. This process involves refluxing the mixture of compounds with carbonyl and amine functional groups [185,187,188]. In the opposite direction, adding water to an imine linkage can lead to hydrolysis; reversing the condensation reaction produces the starting materials (aldehyde and amine) in the presence of an acid catalyst. This process is reversible and reaches thermodynamic equilibrium when reheated [185,187,189,190]. The imine bond is used extensively in various fields due to its advantages. First, it is a reversible bond that can be conveniently synthesized from amine and aldehyde groups. Second, imine compounds are known to have low toxicity levels.

Moreover, amines can be condensed with aromatic aldehydes to produce imines. Condensation reactions between aromatic aldehydes and amines offer notable advantages in organic synthesis. Aromatic imines formed through these reactions exhibit enhanced stability due to resonance stabilization within the aromatic ring, preventing hydrolysis under mild conditions. The regioselectivity of aromatic aldehydes ensures the production of a single desired product, avoiding the formation of unwanted byproducts. Lastly, imines are sensitive to environmental factors, like pH, concentration, and temperature. The sensitivity of imines to environmental factors, like pH, concentration, and temperature, provides significant advantages in organic synthesis. It allows for precise control over reaction conditions, facilitating the fine-tuning of reactivity and selectivity. By adjusting these parameters, chemists can optimize reaction rates and product yields while minimizing the formation of undesired [191].

Hydrogels for various applications have been widely developed using imine bonds due to their reversible association and dissociation behavior, which occurs without intermediates [16,142]. Several recent studies have been conducted on hydrogels based on imine bonds. These studies involved modifying the structural units of polysaccharides using sodium periodate to create aldehyde groups. The oxidized polysaccharides were then used to generate imine bond-based hydrogels with cationic polysaccharides or proteins. Moreover, many biopolymers with primary amine groups, such as gelatin and chitosan, can be used to form imine cross-linking [16,182]. Ding et al. synthesized a hydrogel based on two biopolymers, acrylamide-modified chitosan, and oxidized alginate, using covalent cross-linking to obtain imine cross-links bonds. They found that the cross-linking duration and the pH medium influenced the hydrogels’ mechanical properties and self-healing behaviors. In addition, they expected that these hydrogels could potentially lead to the development of self-healing hydrogels and find a wide range of applications in various fields of biomedicine [192].

Moreover, Ma et al. prepared an injectable hydrogel by combining oxidized alginate hybrid nanoparticles with hydroxyapatite with carboxymethyl chitosan via imine formation. The hydrogels have been observed to exhibit self-healing properties. Additionally, the rheological testing indicates that the storage modulus increases with an increase in the concentration of oxidized alginate or the oxidation time. Given these observations, the hydrogels are promising for bone tissue engineering applications [193]. Lei et al. developed a hydrogel with a self-healing ability based on imine bond formation between gelatin and dialdehyde carboxymethyl cellulose. The gelatin was first reacted with ethylenediamine to increase the amino group content and then cross-linked with dialdehyde groups within the carboxymethylcellulose backbone. The results showed that the hydrogels exhibited good self-healing ability, fatigue resistance, and self-recovery capacity due to the imine bonds [194].

Furthermore, nanocomposite hydrogels based on imine bonds using gelatin/oxidized alginate were prepared by Emami et al. Bisphosphonate-modified hydroxyapatite was incorporated into the hydrogel matrix for bone tissue engineering applications and to improve cytocompatibility. These nanocomposite hydrogels have improved mechanical and rheological properties compared to the hydrogels containing unmodified hydroxyapatite. Additionally, they demonstrated how the bisphosphonate ligand in the oxidized alginate-gelatin cross-links might enhance the dispersion of the nanoparticles via the imine-based reaction. These ligands chelate metal ions on nanoparticle surfaces, anchoring them within the polymer matrix, preventing aggregation, and helping maintain the stability and integrity of the nanoparticles [195].

### 5.1. Particular Cases of Schiff Bases

#### 5.1.1. Hydrazone and Acyl Hydrazone Bonds-Based Hydrogels

Hydrazides are hydrazine derivatives containing a carbonyl or sulfonyl group linked to a nitrogen atom. These compounds retain their specific -NH-NH- nitrogen bridge and have a distinctive terminally occurring hydrazide moiety, which can be represented as R-NH-NH_2_. Hydrazides in imino tautomeric form are a Schiff base example. Hydrazine (H_2_N-NH_2_) can react with aldehydes and ketones to produce a hydrazone derivative. This reaction is a variation of the imine reaction [196].

Hydrazone or acylhydrazone bonds are the most typical reversible reactions, which result from the condensation of hydrazines and hydrazides, respectively, and carbonyl groups (usually a ketone or aldehyde), resulting in the elimination of water [194,195].

The hydrazone bond is characterized by its rapid formation and reversibility under certain conditions and its high stability compared to the imine bond. When the acyl group is introduced into the hydrazide side, the hydrazone bond is transformed into an acyl hydrazone bond. This new bond forms at a slower rate than the hydrazone bond [181,183,196]. Hydrazone or acyl hydrazone reactions are similar to an imine bond, which means they are reversible and can change direction through hydrolysis. Under acidic conditions, the rate of formation or hydrolysis is very fast, which can lead to a rapid exchange of bonds [196,197]. Hydrazone and acyl hydrazone bonds have been widely explored for creating biopolymer hydrogels for various applications based on their unique properties [190]. Hydrazone and acyl hydrazone cross-linking hydrogels are prepared by mixing oxidized polysaccharides with hydrazides or acyl hydrazides. The functionalization of hydrazides is often achieved through carbodiimide chemistry between the free carboxylic acid groups present in the biopolymer backbone and dihydrazide-containing components, such as adipic dihydrazide and carbodihydrazide [16,182]. Scientific researchers have reported hydrogels based on hydrazone/acyl hydrazone cross-links due to their injectable, shear-thinning, and self-healing properties [198,199,200]. Wei and co-workers developed a novel biocompatible self-healing hydrogel by exploiting the dynamic interaction of carboxyethyl chitosan functionalized by adipic acid dihydrazide with oxidized sodium alginate. They demonstrated that the obtained hydrogels exhibit an excellent self-healing ability without the need for any external stimuli, which might be attributed to the dynamic imine and acyl hydrazone bonds in the hydrogel networks.

Moreover, hydrogels show good cytocompatibility and cell release [201]. In another study, Wang et al. confirmed that oxidized hyaluronic acid could be combined with hyaluronic acid functionalized with adipic dihydrazide to generate a self-healing and shear-thinning hydrogel for bioprinting applications. Furthermore, adding the thiol–ene cross-linkable group into the resulting hydrogel networks improves the mechanical properties of the films [202]. Yang et al. prepared a series of cellulose-based self-healing hydrogels constructed through dynamic covalent acyl hydrazone linkages. The synthesized hydrogels display excellent mechanical properties and self-healing abilities with a healing efficiency of over 96%. Additionally, the hydrogels display dual-responsive sol–gel transition behaviors in response to pH and redox, which is promising for the controlled release of doxorubicin [203].

#### 5.1.2. Oxime Bond-Based Hydrogels

**Scheme 1:**
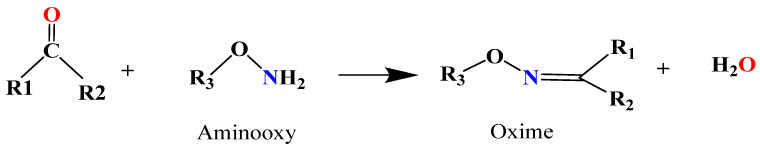
Schematic illustration of Schiff base oxime linkages.

The oxime bond is one of the reversible reactions in dynamic covalent chemistry. It is generated by the condensation reaction of an aldehyde or ketone group and hydroxylamine functional group under mild conditions with water as the only byproduct [181,201,202,203]. Scheme 1 shows a schematic illustration of Schiff base oxime linkages.

As it was mentioned before, oxime formation shows a certain reversibility; however, this reaction offers enhanced hydrolytic stability compared to imine and hydrazine bonds, which causes a limitation in constructing dynamic covalent bulk materials [195,196]. Moreover, the oxime reaction exhibits advantageous characteristics, such as high efficiency, chemo-selectivity, formation in aqueous solvents, and non-toxicity, with water being the only byproduct [204]. Additionally, acidic conditions catalyze the formation of oximes due to electron transfer from the adjacent oxygen [195,202]. Because of the electron-donating resonance effect of the oxygen atom, oximes and oxime ethers are less reactive towards nucleophilic attack than imines because the sp_2_ carbon atom of the oxime is less electrophilic than that of the corresponding imines [205]. Oxime bonds with their specific properties were widely used in several areas, such as in cell surface modification, fabrication of hydrogels, scaffold preparation and conjugation, and the labeling of biological molecules [206]. Recently, oxime cross-linking has been a relatively new promising tool for bioconjugation, especially for the preparation of covalently cross-linked hydrogels. The oxime-based hydrogels are synthesized by incorporating aminooxy-functionalized biopolymers with aldehyde-functionalized biopolymers [182,198]. Mukherjee et al. developed a self-healing oxime-functional hydrogel through reversible gel-to-sol transition in an acidic medium by controlled exchange of oxime functional groups. N, N-dimethyl acrylamide, and diacetone acrylamide were conventionally polymerized to prepare keto-functional copolymers, which were chemically cross-linked with the difunctional alkoxyamines to obtain hydrogels through oxime bonds formation. The workers confirmed that the reversibility nature of oxime cross-links allows the hydrogels to be capable of autonomous healing [207]. In another work, Lin et al. synthesized hydrogels based on the poly(ethylene glycol) (PEG) by the oxime bonds cross-links using dialdehyde-functionalized PEG and a four-arm aminooxy cross-linker molecule. They found that the pH and the presence or the absence of an aniline catalyst influenced the hydrogel properties. Copper(I)-catalyzed alkyne–azide cycloaddition, and metal-free strain-promoted alkyne–azide cycloadditions were also used to generate azide-functionalized oxime hydrogels to include RGD peptides in the hydrogel obtained after gelation to verify the availability of azide groups for post-gelation reactions [206].

Furthermore, 3D photo peptide patterning via thiol–ene chemistry was also investigated in alkene-derived oxime hydrogels. Payam et al. and Cole et al. have demonstrated a synthetic strategy for the preparation of hydrogels using a photolabile protecting group 2-(2-nitrophenyl) propyloxycarbonyl (NPPOC) as a direct photocage for aminooxy group release. The aminooxy groups were released from the NPPOC photocages by UV radiation, and they then interacted with aldehydes derived from (PEG) end-functionalized with benzaldehyde to create oxime linkages. The location and cross-linking degree may be controlled via photo-mediated oxime connections, allowing for the systematic tuning of mechanical properties. Additionally, using photo-mediated oxime linkages, it is possible to immobilize various biomolecules with spatiotemporal control and micron-scale resolution [208,209].

The cross-linking of hydrogels through Schiff base linkages represents a pivotal aspect in developing versatile and functional materials with promising applications across multiple fields. These hydrogels can be effectively cross-linked through various methods, primarily involving imine, hydrazone acylhydrazonation, and oxime linkage formation. The versatility of these cross-linking approaches allows for precise control over the hydrogels’ properties, rendering them adaptable for numerous applications. Despite their significant potential, Schiff base hydrogels face long-term and biodegradability challenges. Researchers are actively working to address these concerns, aiming to enhance the overall performance of these hydrogels. In conclusion, Schiff base hydrogels and their cross-linking methods are promising for developing advanced materials with various applications. However, continuing research is essential to optimize the hydrogel properties and to overcome potential limitations.

## 6. Biomedical Applications of Hydrogels Based on Cross-Linked Proteins with Carbonyl Derivatives of Polysaccharides

Hydrogels have unique properties that make them versatile materials with various engineering applications. Mainly, Schiff base cross-linked hydrogels have gained significant attention in the field of biomedicine due to their biocompatibility, free-standing ability, tissue adhesiveness, porous structure, tunable properties, and potential for controlled drug delivery [210]. These hydrogels form by cross-linking polymeric chains via condensation reactions involving carbonyl and amino groups, forming Schiff bases. The resulting hydrogel structure provides a suitable environment for biomedical applications, such as tissue engineering, drug delivery, wound healing, shape memory, and self-healing systems. Their key advantage is their dynamic nature and ability to adapt to changing conditions, which make them highly versatile and desirable materials for various biomedical applications.

Schiff base metal complexes have the potential to be effective anticancer agents. They can intercalate between DNA base pairs, causing DNA damage and cell death [211]. Although DNA-intercalating anticancer drugs are already in use, new, more potent drugs are necessary due to tumor resistance [212]. Schiff bases provide diverse molecules to screen for anticancer potential. Many Schiff bases have shown promise as anticancer agents through different mechanisms/pathways [213]. Alzheimer’s disease is a condition that affects the elderly population. Beta-peptides are a marker of the progression of the disease. Schiff bases have shown potential in inhibiting the production of beta-peptides and could be used in Alzheimer’s therapy. Schiff bases also have potential in antibacterial therapy, including against tuberculosis. They offer a viable option in the search for new and effective antibacterial agents. The literature on Schiff bases barely scratches the surface of their therapeutic potential. The future will see an expansion in their diverse structures and applications [183].

**Figure 13:**
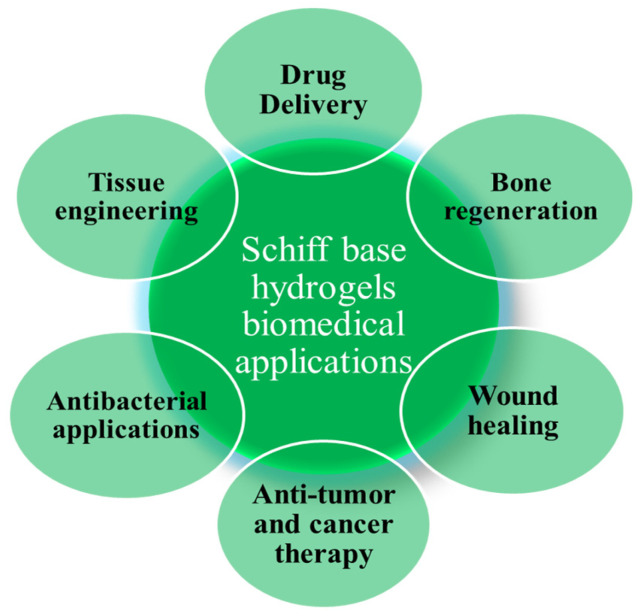
Different biomedical applications of cross-linked hydrogels formed by Schiff base reactions.

**Figure 14:**
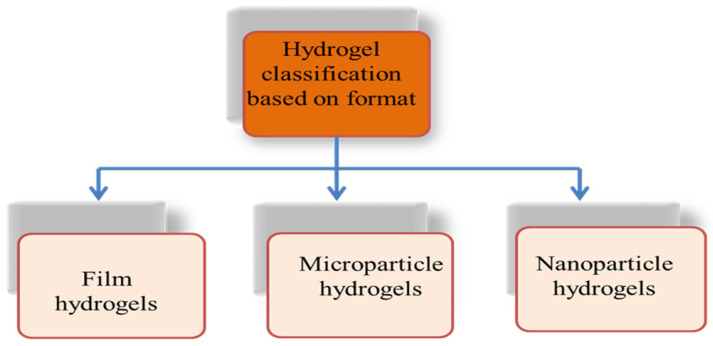
Different formulations of hydrogels based on their format.

Various biomedical applications of hydrogels prepared through cross-linking with Schiff base formation are presented in Figure 13. Depending on the application field, using various techniques, these hydrogels can be prepared in different forms, such as films, membranes, rods, particles, and emulsions [214]. This review discusses the versatility of hydrogels in biomedical contexts, specifically in the form of particles (micro/nanogels) and films as shown in Figure 14. The hydrogel particles provide opportunities for targeted drug delivery, while the hydrogel films can be directly applied to specific sites and have many applications in wound healing and tissue engineering.

### 6.1. Biomedical Applications of Hydrogels Obtained through Schiff Base Chemistry

In recent years, the intersection of polymer chemistry and biomedical sciences has led to remarkable advancements in the field of biomaterials. Among these innovations, microparticles, nanoparticles, and film hydrogels synthesized through Schiff base reactions have emerged as highly versatile platforms with promising applications in healthcare (Figure 13). These hydrogel structures are well-suited for a diverse range of biomedical applications due to the precision and tunability of Schiff base chemistry. From targeted drug delivery and tissue regeneration to diagnostic sensing and wound healing, these hydrogels hold the potential to revolutionize medical therapies and diagnostics, offering a pathway toward more effective and patient-specific interventions.

A recent advancement involves the development of hydrogel films, also known as hydrogel membranes, with precise thicknesses using interconnected networks of natural and synthetic polymers. These hydrogel films inherit the chemical and physical properties of native biopolymers while demonstrating rapid responsiveness and remarkable flexibility. Their compatibility with biological systems and sensitivity to external stimuli make them promising candidates for innovative functionalities. This development opens new directions for various field applications [208,209]. The preparation of hydrogel films based on biopolymers through cross-linking reactions with Schiff base linkage formation is gaining notable attention. This approach offers the potential for various applications due to the combined advantages of biocompatibility, controlled properties of the hydrogel, and the specific features brought about by the Schiff base reaction. These films are particularly valuable in medical and environmental applications, where non-toxic and eco-friendly materials are essential [215,216].

Schiff base hydrogel particles (micro/nanoparticles) are small spherical structures that can be obtained from synthetic or natural polymers and can be prepared in different sizes and shapes by using techniques that are compatible with the encapsulation of biological compounds (for example, cells and drugs) [217]. These biomaterials have a unique potential because they combine hydrogel characteristics (like retaining a high water content) with the characteristics of micro/nanoparticles (small size, large specific surface area). In recent years, hydrogel particles have gained considerable attention in the biomedical field due to their exceptional biocompatibility, significant loading capacity, and responsiveness to environmental factors, such as pH, temperature, and ionic strength [218].

As per the International Union of Pure and Applied Chemistry (IUPAC) definition, a nanogel (hydrogel nanoparticles) is characterized as a gel particle that can take any shape and has an equivalent diameter ranging from 1 nm to 100 nm. On the other hand, a microgel (hydrogel microparticles) has a size diameter between 100 nm and 100 μm [219]. Table 2 represents examples of films and nano/microparticles obtained through covalent cross-linking and Schiff base formation and their biomedical applications.

#### 6.1.1. Biomedical Applications of Schiff Base Hydrogel Films

Schiff base hydrogel films have garnered significant attention due to their unique properties and versatile applications. These hydrogel films exhibit excellent biocompatibility, tunable mechanical properties, and high water absorption capacity. Such characteristics make Schiff base hydrogel films promising candidates for various applications, including drug delivery, wound healing, and tissue engineering.

- ***Drug delivery***

**Figure 15:**
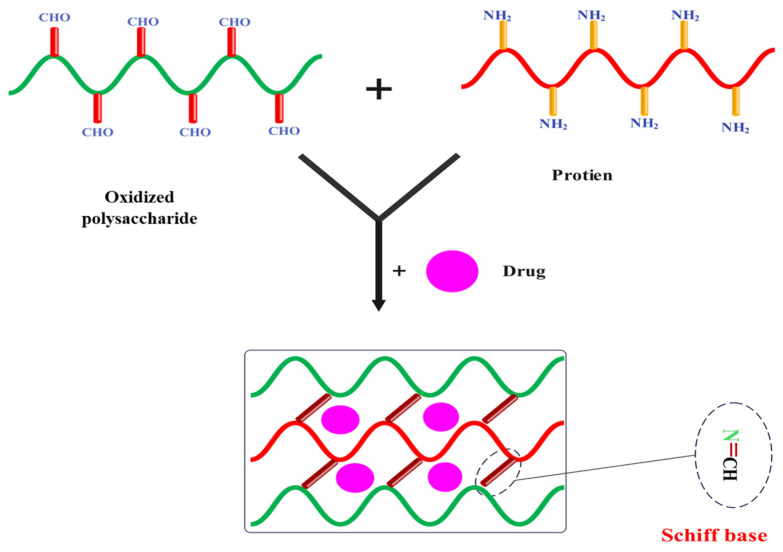
The immobilization of the drug in the film matrix.

Targeted drug delivery involves the precise administration of a drug to a specific location within the body. By utilizing targeted drug delivery, the drug’s efficacy is focused solely on a specified area, thus minimizing potential side effects and ensuring that the viability of cells in that region is not affected [227]. Figure 15 illustrates the immobilization of the drug into the film matrix.

Schiff base hydrogel films have emerged as a promising platform for advanced drug delivery applications. Their unique properties make them particularly well-suited for controlled and targeted drug release. The porous structure of Schiff base hydrogels forms a three-dimensional matrix capable of efficiently encapsulating drugs or bioactive molecules. This polymer matrix protects the encapsulated drugs from degradation, enhancing their stability and enabling controlled release over time.

Additionally, the porosity can be adjusted by modifying the cross-linking density of the hydrogel matrix [2]. The Schiff base hydrogels exhibit high responsiveness to environmental factors, such as pH, temperature, and specific molecules. This property can be utilized to trigger tailored drug release. Moreover, many Schiff base precursors and resulting hydrogels are biocompatible, making them suitable for biomedical applications. Dalei et al. present a compelling argument for the practical application of in situ cross-linked chitosan–dialdehyde guar gum (CsDAGG) hydrogels in dual-drug release strategies for colorectal cancer treatment. The research involved the design of various grades of CsDAGG hydrogels by varying the DAGG content. The study focused on combining curcumin with aspirin to target colon carcinoma. The hydrogels exhibited the ability to protect the drugs from premature absorption in the stomach and small intestine, facilitating a controlled release precisely within the colorectal region [228]. In other studies, poly(vinyl alcohol) (PVA) hydrogels cross-linked with glutaraldehyde (GA) represented an innovative matrix with significant potential in biomedical applications, particularly in ionizing radiation dosimetry. The combination of PVA and GA forms a stable hydrogel system with unique properties that make it suitable for such applications. Locarno et al. conducted a comprehensive study on a hydrogel matrix’s macroscopic and microscopic properties based on PVA chemically cross-linked with GA. The study examined five different kinds of PVAs, varying in molecular weight and degree of hydrolysis. Using ^1^H nuclear magnetic resonance relaxometry, the local microscopic organization of the hydrogels was analyzed. In order to establish correlations with the hydrogel matrix features, the study also investigated different macroscopic properties, including gel fraction, water loss, contact angle, swelling degree, viscosity, and Young’s modulus [229].

Additionally, optical characterization of the hydrogels loaded with Fricke solution was performed to assess their dosimetric behavior. The findings indicate that the degree of PVA hydrolysis is a crucial parameter affecting the structure of the hydrogel matrix, which is essential for ensuring stability over time. This stability is vital for potential biomedical applications where hydrogels serve as radiological tissue-equivalent materials [229]. Moreover, Hosseini and Nabid focused on developing pH-sensitive hydrogel films using basil seed mucilage (OBM) biopolymer as a novel approach to wound dressing and drug delivery. An optimal balance of flexibility and durability was achieved by incorporating varying proportions of PVA, GA as a cross-linker, and glycerol as a plasticizer. The optimized hydrogel film formulations were then utilized to load tetracycline hydrochloride as a model drug, revealing improved release profiles at pH levels 8.5 and 7.4, highlighting their potential for wound healing and drug delivery applications [230].

Additionally, Gallo et al. developed a double network hydrogel combining PVA cross-linked with glutaraldehyde GA and a self-assembling phenylalanine (Phe) peptide to enhance mechanical-elastic properties. They characterized xylenol orange-based Fricke gel dosimeters infused with a Fricke solution within this hydrogel. The dosimeters were irradiated with 6 MV and 15 MV X-rays and analyzed using optical absorbance measurements. The results showed that the double network hydrogel maintained satisfactory radiological water equivalence within the investigated radiotherapy range and exhibited dose rate and energy response independence. The Phe peptide derivative did not hinder the movement of radiation-induced ferric ions, preserving the main characteristics of the standard formulation. The optical response time post-irradiation was similar to traditional PVA-GA dosimeters, with a 30% decrease in radiation sensitivity, which did not impair their use in radiation therapy dose evaluation. The combined dosimetric and mechanical properties make this double network hydrogel promising for biomedical applications as a radiological tissue-equivalent material [231].

- ***Tissue engineering***

Tissue engineering (TE) involves applying scientific and engineering principles to create, regenerate, enhance, or control the structure and functionality of living tissues [232]. TE principles have found widespread use in restoring damaged or dysfunctional tissues and organs, aiming to return them to their normal or improved function [233]. Given the structural resemblance of hydrogels to the extracellular matrix of various tissues, they are employed as scaffolds in regenerative medicine [234]. These scaffolds offer structural support, guide the cellular organization and development, serve as tissue barriers and adhesives, act as drug reservoirs, facilitate the delivery of bioactive substances that promote natural healing, and encapsulate and transport cells. Hydrogels cause favorable tissue responses due to their unique physical and chemical properties. Their high water content makes them highly compatible with cells, allowing for specific or non-specific binding with cellular receptors. Protein and polysaccharide-based hydrogels could incorporate ligands that enhance cell binding, making them valuable scaffolds for cell encapsulation [233]. These biomaterials can undergo chemical or physical cross-linking to obtain various forms of hydrogels, serving as versatile scaffolds for a wide range of regenerative medicine applications.

Schiff base hydrogels possess immense promise across a spectrum of regenerative medicine applications, where meticulous control over scaffold properties plays a pivotal role in ensuring the success of tissue regeneration. In an extensive research effort, Rajalekshmi et al. thoroughly examined the fibrin-incorporated injectable alginate dialdehyde (ADA)–gelatin (G) hydrogel system with potential use as a scaffold for liver tissue regeneration. In order to achieve this objective, they innovatively formulated a hydrogel system by integrating fibrin into ADA-G, which facilitated a comprehensive exploration of its physicochemical and rheological properties. The research included a comprehensive assessment of the hydrogel’s biological characteristics, comprising cytocompatibility, HepG2 cell viability, and the analysis of pivotal functional markers. These assessments covered various aspects, including the indocyanine green uptake assay, live cell imaging analysis (LDL) uptake assay, glycogen storage analysis, CYP-P450 expression analysis, ammonia clearance assay, and albumin assay. The results from these experiments suggest that the fibrin incorporated in ADA-G hydrogel demonstrates the potential to enhance cellular adhesion, proliferation, and overall functionality significantly. These results indicate that the delivery system obtained has excellent potential for use in regenerating liver tissue [235]. Cheng et al. developed a technique to immobilize collagen on cellulose film without causing any conformational changes or degradation. The study investigated the effectiveness of periodate-oxidized regenerated cellulose films in stabilizing collagen through the Schiff base reaction, where NH_2_ groups in collagen interacted with CHO groups in the 2,3-dialdehyde cellulose backbone. The resulting composite material exhibited significant promise as a scaffold for tissue engineering applications. It displayed exceptional strength in a swollen state, offered flexibility in situ, maintained a favorable equilibrium-swelling ratio, allowed for air permeability, and demonstrated remarkable biocompatibility [236]. Liu et al. presented a novel approach involving the synthesis of methacrylate- and aldehyde-functionalized dextran, known as Dex–MA–AD. This work introduced a groundbreaking (or innovative) family of cell-encapsulating hydrogels created by controlling the synergy between the Dex–MA–AD and gelatin polymers. The hydrogel was obtained through ultraviolet (UV)-cross-linking, utilizing methacrylate groups on Dex–MA–AD and a Schiff base reaction between Dex–MA–AD and gelatin. These hydrogels appear promising as 3D vascular tissue engineering scaffolds [237].

In recent work inspired by the lysyl oxidase (LO)-mediated cross-linking process in collagen, Alfei et al. developed a protocol to cross-link gelatin B (Gel B) using polystyrene- and polyacrylic-based copolymers with amine or aldehyde groups. Following slight modifications, they successfully cross-linked Gel B under various conditions, achieving high yields (57–99%) in eight compounds. High cross-linking percentages were observed using styrenic amine (CP5/DMAA) and acrylic aldehyde (CPMA/DMAA) copolymers in the presence of LO. The aerogel-like structure was evident, with densities ranging from 0.10–0.45 g/cm^3^. The best biodegradation profile was noted for the 10% CP5/DMAA, with other samples also showing promising properties. Five composites were identified as suitable for future biological experiments on cell adhesion, infiltration, and proliferation [238].

- ***Wound healing***

Hydrogels used as wound dressings are considered a fundamental element in different wound care approaches, such as superficial burns, abrasions, donor sites, pressure ulcers, and chronic wounds, due to their ability to create an optimal environment for wound cleansing, facilitate the body’s natural removal of necrotic tissue, maintain wound moisture, shield against contamination, absorb exudate, and actively support the healing process [228,235]. Hydrogel treatment offers a multitude of advantages in wound care. It actively promotes the development of new blood vessels and the regeneration of intricate layers of skin, including hair follicles and oil-producing glands, thereby reducing the likelihood of scarring. Furthermore, hydrogels have been employed as a wound dressing to protect against the desiccation of wounds, which is vital for preserving the wound’s moisture content, enhancing patient comfort, helping manage pain, and providing a calming and cooling effect. Thanks to their high moisture content, hydrogel dressings effectively prevent bacteria and oxygen from entering the wound, creating a barrier against infections.

Additionally, hydrogels can promote fibroblast proliferation by reducing fluid loss from wounds, protecting wounds from external irritants, and accelerating the healing process. Hydrogel wound dressings also help maintain an optimal microclimate for cellular activities on the wound surface, which is essential for biosynthetic reactions [228,235,236,237].

Schiff base hydrogel films are essential in maintaining a moist wound environment, a crucial factor in facilitating effective wound healing. They are generally well-tolerated by the skin and are biocompatible, which reduces the chance of irritation or allergic reactions [239]. One significant benefit of these films is that they are non-adherent when in contact with the wound, minimizing the risk of trauma or damage to newly-formed tissue during dressing changes [240].

Depending on their composition, Schiff base hydrogels may also possess inherent antimicrobial properties, increasing their effectiveness in preventing wound infections [241,242,243]. Their simplicity in application and capacity to conform to diverse wound shapes and sizes serve to improve their attractiveness even more.

Guo et al. conducted a new study focusing on developing a hydrogel used as a wound dressing with substantial potential for chronic wound healing applications. Their innovative hydrogel was obtained through a Schiff base reaction using oxidized hyaluronic acid (OHA) and carboxymethyl chitosan (CMCS). This hydrogel was further enriched by incorporating active polypeptides extracted from Periplaneta Americana (PAE), an American cockroach (The role of the peptide was likely to enhance the hydrogel’s properties or functionality. Peptides extracted from natural sources, like Periplaneta Americana, often possess bioactive properties, such as antimicrobial, antioxidant, or wound-healing effects. Therefore, incorporating these peptides into the hydrogel may have conferred additional therapeutic benefits, such as promoting tissue regeneration or providing antimicrobial protection). OHA/CMCS/PAE composite hydrogels are promising candidates for addressing chronic wound-healing challenges. These hydrogels, with their robust properties and demonstrated therapeutic effects, offer a potential way to improve the management of chronic wounds, particularly in diabetic wound-healing scenarios [67]. In a separate study, Zhang et al. employed a Schiff base reaction to prepare hydrogels using oxidized sodium alginate, chitosan, and zinc oxide. These hydrogels exhibited a remarkable degree of swelling and porosity. Rigorous in vitro experiments assessed their biocompatibility with diverse cell lines, encompassing 293T cells, blood cells, and 3T3 cells. The findings were quite encouraging, showcasing the hydrogels’ favorable biocompatibility. Notably, these hydrogels demonstrated exceptional antibacterial properties, effectively combating *Bacillus subtilis*, *Candida albicans*, and *Staphylococcus aureus*. Simultaneously, they exhibited the capacity to accelerate the healing process of scalded wounds in rat models. This comprehensive study underscores the potential and versatility of these hydrogels in various biomedical applications [244].

Furthermore, in a study by Oh et al., hydrogels were obtained through a Schiff base reaction involving gelatin, oxidized sodium alginate, chitosan, and salicylic acid. These hydrogels exhibited remarkable wound-healing properties and demonstrated a notable absence of toxicity [245].

#### 6.1.2. Biomedical Applications of Hydrogel Microparticles Containing Schiff Bases

In biomedical applications, microgels, which are hydrogel particles characterized by their micron-scale diameters, play an essential role. Their small size facilitates injection and the possibility of modifying their surface, making them versatile tools in the biomedical field. With an extensive specific surface area and a remarkable loading capacity, microgels hold promises for localized therapeutic delivery and integration into granular scaffold structures. The hydrogel microparticles have numerous biomedical applications, such as topical drug delivery, bone and soft tissue regeneration, and immunomodulation. Similarly, hydrogel microparticles obtained through cross-linking with imine bond formation are essential in biomedical applications. These hydrogels offer tunable properties, including responsiveness to environmental factors, biocompatibility, and the ability to control the release of encapsulated biological molecules. Their capacity for tailored responses to physiological conditions enables precise drug targeting. Furthermore, their biocompatibility ensures harmonious interactions with living tissues, rendering them ideal for tissue engineering and regenerative medicine applications. The potential impact of Schiff base particle hydrogels on the biomedical field is significant, promising enhanced drug delivery precision, tissue regeneration support, and therapeutic strategy advancements.

- ***Drug delivery applications***

Hydrogel microparticles are a popular drug delivery system. Bioactive agents are either physically bound to the delivery system or encapsulated in hydrogel microparticles [184,246,247]. Compared to conventional hydrogels, they can immobilize various drugs, proteins, and nucleic acids [247].

The application of the Schiff base reaction, which involves the interaction between a polysaccharide, typically containing amine and aldehyde functional groups, has been involved in the development of various hydrogels and microgels with relevance to biomedical contexts. The literature reports on the advantages of hydrogels synthesized via Schiff base chemistry, including their biocompatibility, antibacterial properties, and responsiveness to environmental factors [248]. Du et al. developed drug-loaded microgels through the Schiff base reaction involving carboxymethyl chitosan and oxidized carboxymethyl cellulose. These microgels were then incorporated into hydrogels, obtaining a remarkable hydrogel–microgels composite noted as Gel/MGs. The results confirmed that incorporating microgels conferred superior stability, improved mechanical strength, and increased drug release sensitivity to both acidic and alkaline conditions in vitro [248].

Additionally, Gel/MGs wound dressings exhibit desirable antibacterial properties, making them promising candidates for wound dressing applications [248]. Su et al. developed hydrogel microparticles by forming Schiff bases between aldehyde dextran and ethylenediamine within the water-in-oil (W/O) microemulsion system. These microgels had a particle size ranging from 800 to 1100 nm. Su et al.’s pioneering work highlights the potential of pH-sensitive microgels as versatile and efficient drug delivery systems, holding great promise for various biomedical applications [218]. Yan et al. have recently ingeniously constructed hydrogel microparticles tailored for pulmonary drug delivery. These microparticles, comprising Zn^2+^, carboxymethyl chitosan, and hyaluronan aldehyde, were prepared using the spray drying technique. The results showed that the obtained hydrogel microparticles exhibited a sustained drug release profile, reaching the equilibrium after 24 h in vitro. They effectively avoided being cleared by alveolar macrophages and had significantly longer residence times in vivo, making them potentially useful for improving the treatment and management of respiratory conditions [249]. In another study, Cheng et al. presented an innovative injectable hydrogel microparticle for obtaining a co-delivery system designed for the sustained release of antibiotics, namely lysostaphin and vancomycin, at the infection sites. The two-step process involved microfluidic encapsulation of Van within gelatin methacryloyl microgels followed by incorporation into a gelatin and oxidized starch prepolymer solution to form a hydrogel through a Schiff base reaction. The addition of transglutaminase created a dual cross-linked network scaffold for the controlled release of the drugs. This system offers sequential antibiotic release, enabling targeted biofilm bacteria eradication and minimal toxicity from hydrogel degradation products, promising a practical approach to infection treatment [250,251].

- ***Cell encapsulation***

Hydrogel microparticles, specifically Schiff base hydrogel microparticles, have gained considerable attention as a versatile platform for cell encapsulation in various biomedical applications. These tiny, gel-based particles are engineered to incorporate and protect individual cells or cell clusters, providing an ideal microenvironment for their growth, proliferation, and therapeutic activity. The hydrogel matrix of these microparticles can be precisely tailored to mimic the extracellular matrix, offering mechanical support, nutrient exchange, and the controlled release of bioactive molecules [172,212]. Jang et al. designed a polysaccharide-based microgel using Schiff base cross-linking between oxidized dextran (ODX) and N-carboxyethyl chitosan (N-CEC). NIH-3T3 fibroblast cells (mouse embryonic fibroblast cell line) were encapsulated within the ODX/N-CEC-based microgels, and the viability of the cells was further analyzed. The resulting microgels had high biocompatibility and represent a promising approach for creating on-the-spot microgels from two viscous polymer solutions, offering versatility for various biomedical applications [252].

- ***Bone regeneration applications***

The process of bone regeneration is complex and demands novel approaches to improve its effectiveness. Microparticle Schiff base hydrogels (MSBHs) are a promising advancement in this field. What makes MSBHs unique is their microparticulate structure and properties, such as softness, size, injectability, porosity, and degradability. These qualities make them suitable for carrying drugs, bioactive factors, and cells, which can be designed for tissue repair and regeneration [253]. Zhou et al. have obtained an innovative injectable system using gelatin–methacryloyl (Gelma) hydrogel microspheres as the delivery system. They aim to induce epigenetic reprogramming within microfluidic microsphere systems to enhance bone regeneration and modulate the microenvironment during the initial phase of fractures. This system is designed for macrophage targeting and uses micro/nano microspheres of Gelma phosphatidylserine-specific liposomes (Gelma@Lip@Pla) for enhanced therapeutic effects. The required Gelma@Lip@Pla microgel was formed by Schiff base cross-linking between the residual amino group on the surface of the low-substituted Gelma and the aldehyde group on the surface of the Lip@Pla. The researchers confirmed that the Gelma@Lip@Pla microgel exhibits significant promise as an extended-release platform for addressing bone defects and various immune-related conditions that involve macrophages [254]. In their research, Zhang et al. created a 3D microsphere composed of bacterial cellulose and collagen, joined through Malaprade and Schiff base reactions, with a multi-stage structure and composition. The goal was to use it in bone tissue engineering applications. This multi-level structure exhibited excellent biocompatibility and significantly promoted the attachment, growth, and osteogenic differentiation of MC3T3-EL cells in mice [255]. These Schiff base-linked “smart” hydrogels not only provide protection for encapsulated drugs, peptides, and proteins against the external environment but also enable controlled release by responding to changes in the polymer network’s swelling and shrinking, effectively acting as an “on-off” switch for controlled drug delivery [69].

#### 6.1.3. Biomedical Applications of Hydrogel Nanoparticles (Nanogels) Obtained by Schiff Base Reaction

Nanogels have various advantages, such as a stable size, excellent hydrophilicity, biocompatibility, and adaptability to specific environmental conditions. They have minimal non-specific interaction with blood proteins, reducing the likelihood of immune response. Nanogels are promising competitors in the field of biomedicine. In this section, we provide a brief overview of the recent applications of nanogels for drug delivery, anti-tumor/cancer treatments, antibacterial applications, nerve regeneration, disease prevention, and diagnosis.

- ***Drug Delivery Applications***

Numerous nanogels exhibit exceptional drug encapsulation efficiency and loading capacity, making them suitable for transporting a wide range of pharmaceuticals and encapsulating hydrophilic and hydrophobic drugs. Moreover, the nanogel’s structure can be easily tailored to incorporate characteristics from various materials, providing a significant advantage for the simultaneous encapsulation of drugs with diverse physicochemical properties, including small molecules, proteins, and nucleic acids [256].

Schiff base nanogels have a unique ability to create and break reversible covalent bonds. This ability enables the maintenance of a stable drug structure while allowing for controlled and targeted drug release. Scientists can engineer these nanogels to respond to specific environmental factors, such as changes in pH, temperature, or enzymatic activity, which helps them to customize the drug release profiles. This customization is beneficial for different therapeutic applications. In a pioneering study by Su et al., they obtained a dextran-based nanogel system that ingeniously improved the strength of Schiff base formation for drug delivery, specifically employing doxorubicin (DOX) as the model drug. This innovative nanogel was synthesized using the inverse microemulsion technique, where covalent conjugation of DOX to the nanogel was achieved via Schiff base linkages. This nanogel has a pH-dependent drug release profile due to its acid-sensitive Schiff base linkages. Drug release is faster in acidic media compared to in a physiological solution. This research demonstrates the integration of nanotechnology and chemistry to develop intelligent drug-delivery systems [257]. In a notable study, Sarika et al. encapsulated curcumin, a natural compound with therapeutic potential, within nanogels composed of dialdehyde alginate and gelatin. They employed the inverse miniemulsion technique to obtain the nanoparticles. The results of their research undoubtedly highlight the effectiveness of ingeniously designed nanogels for delivering curcumin specifically to breast cancer cells. This promising development opens new opportunities for more efficient and targeted drug delivery in breast cancer treatment [258].

- ***Anti-tumor and cancer therapy***

Schiff base nanogels are emerging as promising delivery systems in the field of anti-tumor and cancer therapy. These nanogels offer a versatile platform for drug delivery with a particular focus on targeting cancer cells. Nanogels can be created using the Schiff base chemistry method and can be designed to react to specific pH levels commonly present in tumor microenvironments. This pH sensitivity enables the nanogels to release drugs in a controlled and selective manner at the tumor site, thus reducing off-target effects and improving therapeutic efficacy [259]. One of the notable advantages of Schiff base nanogels is their ability to encapsulate a wide range of anti-cancer drugs, including chemotherapeutic agents, targeted therapies, and even macromolecules, like nucleic acids. This versatility enables tailored treatment approaches for different types and stages of cancer [254,257]. Moreover, Schiff base nanogels with multiple aldehyde groups provide opportunities for further functionalization, such as the conjugation of targeting ligands or imaging probes. This enhances the specificity of drug delivery to cancer cells and reduces collateral damage to healthy tissues [260].

As research in this area continues to evolve, Schiff base nanogels hold significant promise for revolutionizing cancer treatment strategies. Yu et al. synthetized dextran-based nanogels using a direct method that involved the formation of disulfide intermolecular bonds and Schiff base linkages between polyaldehyde dextran and cystamine. The process was carried out in a water-in-oil inverse microemulsion. The distinctive feature of these nanogels lies in their responsiveness to both acidic and reductive environments, a crucial feature for drug delivery in cancer therapy. The ingenious aspect of this research was the covalent conjugation of the anticancer drug DOX into the dextran nanogels via Schiff base linkages. This strategic drug encapsulation allowed for the development of pH/GSH (glutathione) dual-responsive drug release profiles, ensuring that DOX would be released predominantly in the acidic and reductive tumor microenvironment [261].

Bashiri et al. used gum Arabic oxidized with sodium periodate as a naturally derived, non-toxic cross-linker for the fabrication of hybrid bovine serum albumin–gum Arabic aldehyde nanogels through a Schiff base reaction using 5-fluorouracil as the anti-cancer model drug. The entire synthesis process was conducted without using toxic organic solvents, with fractionated coconut oil as the continuous phase. The study’s outcomes indicated that these biobased hybrid nanogels are promising for various anti-cancer therapies [226]. Ziaei et al. developed pH-responsive, in situ forming hydrogels using oxidized alginate and gelatin with encapsulated DOX in chitosan/gold nanoparticles nanogels. The polymer chains were interconnected through Schiff base bonds, and beta-glycerophosphate was used as an ionic accelerator cross-linker. As anticipated, both the drug-loaded hydrogel and free DOX at a specific concentration led to significant cell death in MCF-7 cells, highlighting the potential of these hydrogels for localized breast cancer treatment [262].

- ***Antibacterial Applications***

Bacterial biofilm formation poses a significant challenge in clinical practice, often leading to implant failure and life-threatening complications. Addressing this issue is crucial, and nanogels offer a promising solution due to their stability and drug-targeting abilities. These nanogel carriers enable the controlled release of antimicrobial agents at high concentrations in localized areas, making them valuable tools in combating bacterial infections and their complications [214,260]. Reactive oxygen species (ROS) have also shown significant antimicrobial action against a wide range of Gram-positive and Gram-negative organisms, including multidrug-resistant isolates and biofilm-producing pathogens. Utilizing ROS as a therapeutic approach for topical applications on the skin, mucosal membranes, or internal tissues colonized by microbial inhabitants and biofilms offers a novel strategy to enhance infection control and improve clinical outcomes [263].

Moreover, integrating Schiff base chemistry in nanogels enhances their versatility for antibacterial applications. The Schiff base linkages within these nanogels can be tailored to respond to specific environmental factors, which shows great potential in combating bacterial biofilms and could revolutionize antibacterial and anti-infection strategies in clinical settings. Recently, Chung et al. engineered nanogels (NGs) through precise chemical modifications. They cross-linked aldehyde groups with primary amines using a Schiff reaction to create DNA-HCl-S-benzyl-L-cysteine (SBLC). Simultaneously, alginate acid (AA) was treated with a water-soluble derivate of carbodiimide (EDAC), leading to AA-EDAC, which was further substituted with SBLC to produce AA-SBLC. These modifications were integrated into an emulsification process, resulting in spherical NGs with grafted benzene rings. The authors confirmed that SBLC modifications significantly improved their antimicrobial properties. This study offers a promising clinical solution for antibiotic-resistant biofilm strains [264]. In another study, Gao et al. fabricated antimicrobial quaternized chitosan/Ag composite nanogels (QCS/Ag CNGs) using an inverse miniemulsion technique with a high encapsulation efficiency of NH_2_-Ag nanoparticles. The QCS/Ag CNGs display potent broad-spectrum antimicrobial properties with minimal toxicity, achieved through the combined action of Ag nanoparticles and QCS. These NH2-Ag NPs are securely bound to the QCS structure through Schiff base reactions, and the resulting QCS/Ag CNGs possess reactive groups, ensuring long-lasting antibacterial cotton fabrics. This research presents a straightforward and adjustable approach for producing polymer/inorganic CNGs, offering a solution to the pressing demand for antibacterial materials and fabrics [265].

## 7. Conclusions

Hydrogels, derived from polysaccharides cross-linked with proteins, have garnered significant attention due to their remarkable properties and potential biomedical applications. Modifying polysaccharides by introducing carbonyl groups can fine-tune their properties, enhancing their functionality for specific applications. This modification has led to advancements in targeted drug delivery and biomaterial improvement.

Chemical oxidation methods precisely control this modification, resulting in functionalized polysaccharides that are better suited for advanced biomaterials development. In hydrogel preparation, adding carbonyl groups to polysaccharide backbones improves the hydrogel’s mechanical properties and responsiveness to external stimuli. The Schiff base method is used to cross-link modified polysaccharides with proteins, creating versatile hydrogels tailored for specific biomedical applications. Injectable hydrogels, films, beads, macroscopic structures, and nanoparticles find unique utility in drug delivery, tissue engineering, and wound healing, positioning Schiff base hydrogels as promising biomaterials for various biomedical applications.

## 8. Perspectives

Hydrogels made from proteins cross-linked with carbonyl derivatives of polysaccharides are an exciting area of study in biomaterials research with significant potential for biomedical applications. These hybrid hydrogels combine the beneficial characteristics of proteins and polysaccharides, providing biocompatibility, biodegradability, and customizable properties. Effective cross-linkers, such as aldehyde-modified chitosan and oxidized alginate allow precise control over mechanical strength, swelling behavior, and degradation rates. These hydrogels are particularly promising for tissue engineering, drug delivery, and regenerative medicine, serving as scaffolds, delivery systems, and implantable devices. Their biodegradability reduces long-term adverse effects, and their ability to encapsulate bioactive molecules enhances therapeutic outcomes.

However, several challenges and opportunities remain. In order to achieve consistent and scalable hydrogel production, it is essential to optimize the synthesis methods, including cross-linking efficiency and gelation kinetics. Additionally, comprehensive characterization and evaluation of their biocompatibility, mechanical properties, and degradation behavior are crucial to ensure their safety and effectiveness in clinical trials. Moreover, exploring new formulations, such as hybrid hydrogels with enhanced responsiveness to stimuli or multifunctional capabilities, has the potential to address unmet needs in regenerative medicine and drug delivery.

From the chemical technology and material engineering perspective, a critical approach is necessary to address these solutions’ limitations and low market availability. The synthesis of these hydrogels can be complex and resource-intensive, posing challenges for large-scale production. Additionally, the variability in natural polysaccharides and proteins can lead to inconsistencies in hydrogel properties, necessitating stringent quality control measures. Market availability is further limited by the high costs associated with advanced materials and the regulatory hurdles for biomedical applications. Overcoming these obstacles requires ongoing innovation in chemical synthesis techniques, improved standardization protocols, and collaborative efforts between researchers, the industry, and regulatory bodies.

## Figures and Tables

**Table 1:** Examples of oxidized polysaccharides and their properties.

Polysaccharide	Oxidizing Agent	Oxidized Polysaccharides Properties	References
**Sodium Alginate**	Sodium periodate	Low Mw with the increase in periodate dosage. The aldehyde group content was higher when the periodate concentration increased. The cross-linking with the collagen was enhanced as oxidized polysaccharides’ molecular weight (Mw) decreased.	[50]
	Sodium periodate	Alginate extensive cleavage was observed in the ethanol–water mixture. In the aqueous solution, the Mw decreased gradually with the increase in periodate concentration. In the ethanol–water mixture, the change in Mw was not correlated with an increase in periodate content.	[82]
	Sodium periodate	The molar weight (Mw) decreased until an oxidation of 10 mol%. Low capacity to form gels with calcium ions beyond the oxidation of 10 mol%.	[83]
**Chitosan**	Periodate	A pronounced increase in chain flexibility.	[98]
	Periodate	Chitosans became only partially oxidized by periodate, achieving degrees of oxidation around 0.5. The rate and extent of overoxidation, as well as the rate of depolymerization, decreased when increasing the fraction of remaining N-acetylated residues (N-acetyl-D-glucosamine).	[99]
**Polullan**	Sodium periodate	The highest oxidation degree and dialdehyde group content were observed at pH 4.0. The addition of dialdehyde pullulan significantly improved the mechanical properties of gelatin hydrogels.	[108]
	Sodium periodate	Periodate oxidation of pullulan was used to cross-link gelatin edible films.	[102]
	Sodium periodate	Pullulan was oxidized using sodium periodate to create its aldehyde derivative, which was utilized as a bio cross-linker for stabilizing collagen.	[104]
**Carboxymethyl cellulose**	Sodium periodate	The oxidation method substantially degraded the carboxymethyl cellulose structure. A decrease in the hydrodynamic radius and crystallinity of carboxymethyl cellulose.	[109]
	Sodium periodate	Low thermal stability of the oxidized carboxymethyl cellulose at the increased aldehyde contents. Reduction in the crystallinity becomes significant when the aldehyde content within the polysaccharide structure is higher.	[115]
	Sodium periodate (first method) Hydrogen peroxide in the presence of iron tetrasulfophthalocyanine (second method)	The first method is the most promising for further research.	[111]
**Pectin**	Periodic acid	Low intrinsic viscosity. A high aldehyde content is obtained at high temperatures and when the concentration of periodic acid increases. More significant aldehyde contents were obtained in the water–ethanol system than in a pure aqueous medium. An increase in the ethanol content increased the number of moles of the aldehyde group formed.	[121]
	Sodium periodate	Low intrinsic viscosity. Low molecular weight.	[126]

**Table 2:** Representative examples of hydrogel forms obtained through covalent cross-linking and Schiff base formation and their biomedical applications.

Hydrogel Type	Cross-Linking Types	Materials	Biomedical Application	References
**Film**	Imine bond	Polymers of acrylamide-modified chitin (AMC) containing amino groups and oxidized alginate	Various biomedical fields	[216]
**Microgel**	Enzymatic cross-linking	Gelatin–gelatin (using transglutaminase (mTG))	Wound healing and tissue engineering	[220]
**Microgels**	Enzymatic cross-linking and Diels–Alder reaction	Furyl amine (furan) and tyramine (TA) grafted hyaluronic acid (HA) molecules (HA–furan/TA)	Cell encapsulation and delivery	[221]
**Microgel**	Hydrazone cross-linking	Hyaluronic acid hydrazide–hyaluronic acid aldehyde Hyaluronic acid hydrazide–poly(ethylene glycol) dialdehyde	Treating vocal fold scarring, not just as biocompatible filler materials; repairs focal defects, smooths the vocal fold margin, and potentially softens and dissolves scar tissue	[222]
**Microgels**	Hydrazone cross-linking	Hydrazide functionalized carboxymethyl cellulose–aldehyde functionalized dextran (DEX-B)	Cell encapsulation or drug delivery	[223]
**Microgel**	Imine cross-linking	Oxidized sodium alginate–chitosan	Polymeric system for the colon-specific delivery of anti-inflammatory drugs, including 5-ASA’ enhances the therapeutic effect of ulcerative colitis	[224]
**Nanogel**	Thiol–ene coupling	Hyaluronic acid–poly(ethylene glycol)–bis(thiol)	Drug delivery	[225]
**Nanogel**	Imine cross-linking	Hybrid bovine serum albumin–arabic gum aldehyde (BSA-GAA)	Drug delivery and other biomedical applications	[226]

## Data Availability

The data presented in this study are available on request from the corresponding authors.
